# Whole-genome resequencing reveals domestication and signatures of selection in Ujimqin, Sunit, and Wu Ranke Mongolian sheep breeds

**DOI:** 10.5713/ab.21.0569

**Published:** 2022-04-29

**Authors:** Hanning Wang, Liang Zhong, Yanbing Dong, Lingbo Meng, Cheng Ji, Hui Luo, Mengrong Fu, Zhi Qi, Lan Mi

**Affiliations:** 1 State Key Laboratory of Reproductive Regulation and Breeding of Grassland Livestock, School of Life Sciences, Inner Mongolia University, Hohhot 020020, China; 2 Hebei Provincial Key Laboratory of Basic Medicine for Diabetes, The Shijiazhuang Second Hospital, Shijiazhuang 050051, China

**Keywords:** Domestication, Metabolism, Sheep, Selection, Whole-genome Resequencing

## Abstract

**Objective:**

The current study aimed to perform whole-genome resequencing of Chinese indigenous Mongolian sheep breeds including Ujimqin, Sunit, and Wu Ranke sheep breeds (UJMQ, SNT, WRK) and deeply analyze genetic variation, population structure, domestication, and selection for domestication traits among these Mongolian sheep breeds.

**Methods:**

Blood samples were collected from a total of 60 individuals comprising 20 WRK, 20 UJMQ, and 20 SNT. For genome sequencing, about 1.5 μg of genomic DNA was used for library construction with an insert size of about 350 bp. Pair-end sequencing were performed on Illumina NovaSeq platform, with the read length of 150 bp at each end. We then investigated the domestication and signatures of selection in these sheep breeds.

**Results:**

According to the population and demographic analyses, WRK and SNT populations were very similar, which were different from UJMQ populations. Genome wide association study identified 468 and 779 significant loci from SNT vs UJMQ, and UJMQ vs WRK, respectively. However, only 3 loci were identified from SNT vs WRK. Genomic comparison and selective sweep analysis among these sheep breeds suggested that genes associated with regulation of secretion, metabolic pathways including estrogen metabolism and amino acid metabolism, and neuron development have undergone strong selection during domestication.

**Conclusion:**

Our findings will facilitate the understanding of Chinese indigenous Mongolian sheep breeds domestication and selection for complex traits and provide a valuable genomic resource for future studies of sheep and other domestic animal breeding.

## INTRODUCTION

Domestication reshaped animal morphology, physiology, and behavior, providing the opportunity to study the molecular processes triggering evolutionary change. Animal domestication has been widely studied to understand the animal genetic and phenotypic alterations in various species including pig [1], cattle [2], and sheep [3,4]. Sheep (*Ovis aries*) is one of the most important livestock that provide meat, fur, and sheep milk for humans. The history of Chinese sheep domestication is traced back over 5,000 years [3,4]. Chinese sheep originated from the Mongolian Plateau about 5,000 to 5,700 years ago. Afterwards, they might spread in the upper and middle reaches of the Yellow River about 3,000 to 5,000 years ago (3 to 5 kya), and reach the Qinghai-Tibetan and Yunnan-Kweichow plateaus about 2 to 2.6 kya [5]. Up to now, about 42 native sheep breeds have been harbored in China [6].

Mongolian sheep evolved from the wild Argali sheep in Central Asia area, migrated to northern parts of China, and were introduced into other provinces including Gansu, Shandong, Qinghai [7,8]. Therefore, most of modern Chinese sheep breeds such as Hu sheep and Small-tailed Han sheep have a close relationship to Mongolian sheep [8]. Inner Mongolia grassland is in the north of China. The northern grassland ecosystems of China play key roles in supporting a variety of species of plants and animals [9]. In Inner Mongolia regions, Mongolian sheep are distributed in various areas. For instance, Hulunbuir sheep are mainly in Hulunbuir Grassland. In Xilingol Grassland, there are three different grazing Mongolian sheep including Ujimqin sheep (UJMQ), Sunit sheep (SNT), and Wu Ranke sheep (WRK), which are famous for their excellent meat quality.

To characterize genetic variations involved in domestication and genetic improvement, whole-genome resequencing has been performed and deeply analyzed in multiple organisms. In sheep, genomes with Asiatic mouflon and Chinese native sheep and candidate genes responsible for traits have been investigated [6,10]. For instance, various sheep adapted to different ecological environments including dry and humid climates and high-altitude hypoxia were explored to identified functional genes and genetic variation [11–13]. Genes with tail fat deposition and short-tail phenotype were also revealed based on genome sequencing of several breeds sheep such as Hulunbuir sheep, UJMQ, Tan sheep, Small-Tail Han sheep, Cele Black Sheep, and Hu sheep [14–16]. Recently, deep resequencing of sheep including Asiatic mouflon, landraces, and improved breeds was performed and genes related to morphological and agronomic traits were thus identified [17]. However, the domestication and genetic similarities and differences of these three sheep remain unclear.

In this study, we performed whole-genome resequencing of Mongolian sheep breeds including WRK, UJMQ, and SNT distributed in different regions around Xilingol Grassland locates in the north of China to clarify the overall genetic variation, population structure, domestication, and selection for domestication traits among these Chinese local Mongolian sheep breeds. These findings will contribute to the understanding of the demographic history of Chinese indigenous Mongolian sheep breeds, and provide a genetic resource of great value for studying sheep and other domestic animals breeding in the future.

## MATERIALS AND METHODS

### Sample collection, DNA extraction, and sequencing

To obtain blood from purebred WRK, UJMQ, and SNT, we went to the local breeding sheep farm and collected sheep blood samples. Blood samples were collected from a total of 60 individuals comprising 20 WRK, 20 UJMQ, and 20 SNT. Wu Ranke 4-year-old female breeding sheep were from the Wu Ranke breeding sheep base in the Bayan oboo Gacha, Jiergalangtu Sumu, Abaga Banner, Inner Mongolia. Ujimqin 2-year-old female breeding sheep were from the Ujimqin breeding sheep farm of East Ujimqin Banner Dongxing Livestock comprehensive development base in Amuguleng Gacha, Wuliyasitai town, East Ujimqin Banner, Inner Mongolia. Sunite 2-year-old female breeding sheep were from the Sunite breeding sheep farm of Sonid Left Banner in Bayan Erdene Gacha, Dalai Sumu, Sonid Left Banner, Inner Mongolia. For genome sequencing, about 1.5 μg of genomic DNA was used for library construction with an insert size of about 350 bp. Pair-end sequencing were performed on Illumina NovaSeq platform (Illumina, San Diego, CA, USA), with the read length of 150 bp at each end. The resequencing data have been submitted to the SRA database in NCBI with SRA: SRP338713.

### Alignment and variation calling

Raw reads performed quality control with Trimmomatic (v0.36) [18] and filtered high quality reads were aligned against the sheep reference genome (Oar_v3.1) using BWA-MEM (version 0.7.12) with default parameters [19]. Single nucleotide polymorphisms (SNPs) and insertion and deletion (INDELS) were called using the Unified Genotyper implemented in GATK (v4.1.2.0) and subsequently filtered using the hard filtering process recommended by GATK [20]. For each locus, the genotype with maximum posterior probability was picked as the genotype for that locus. Variations were annotated with the ENSEMBL Variant Effect Predictor (VEP v98) [21].

### Genome-wide association study

Variation and phenotype data were analyzed by PLINK, and phenotypic information including demographic information, age, sex, and breeds, which from a group of individuals. The variants with a minor allele frequency of less than 0.05, missing call frequencies greater than 0.1 and Hardy-Weinberg equilibrium exact test p-value less than 0.00001 were excluded. Then pairwise genome-wide association analysis was performed between three breeds of sheep with PLINK, p-value was adjusted by Bonferroni method. Genome-wide association study (GWAS) results visualized with R package rMVP [22].

### Population genetics analysis

The neighbor-joining tree was constructed with filtered variations for all samples based on the nucleotide p-distance matrix then visualized using R package ggtree. Principal component analysis (PCA) analysis was performed with PLINK v1.9 and the first 2 eigenvectors were used for visualizing the results. Population structure was assessed by ADMIXTURE v1.3.0 with default parameters [23]. Furthermore, we used PSMC method to estimate the changes in the effective population (Ne) of sheep over the last 10,000 years [24]. The parameters were set as follows: -N30 -t15 -r5 -p ′4+25×2+4+6′. An average mutation rate (l) of 2.5×10^−8^ per base per generation and a generation time (g) of 3 years based on previous study [17]. Bootstrapping was performed 100 times for each sample.

### Selective sweep analysis

Tests for selective sweeps analysis was performed based on *F*st (Fixation index), pairwise single-site nucleotide diversity (*θ*π) and Tajima’s D between three breeds of sheep via vcftools (v0.1.15) [25]. *θ*π ratios (*θ*πSNT/*θ*πUJMQ, *θ*πSNT/*θ*πWRK, and *θ*πUJMQ/*θ*πWRK) of those three breeds were calculated with a sliding window approach (100 kb windows with 10 kb increments), and the *θ*π ratios were log_10_-transformed. Tajima’s D value was also calculated with a sliding-window approach (100 kb windows with 10 kb increments) to assess the selective signature for target genes [26]. The gene ontology (GO) functional and Kyoto encyclopedia of genes and genomes (KEGG) pathway enrichment analyses of genes with significant signals in GWAS and selective sweep analysis were performed in R with GOstats package [27].

## RESULTS

### Genetic variation

**Figure 1:**
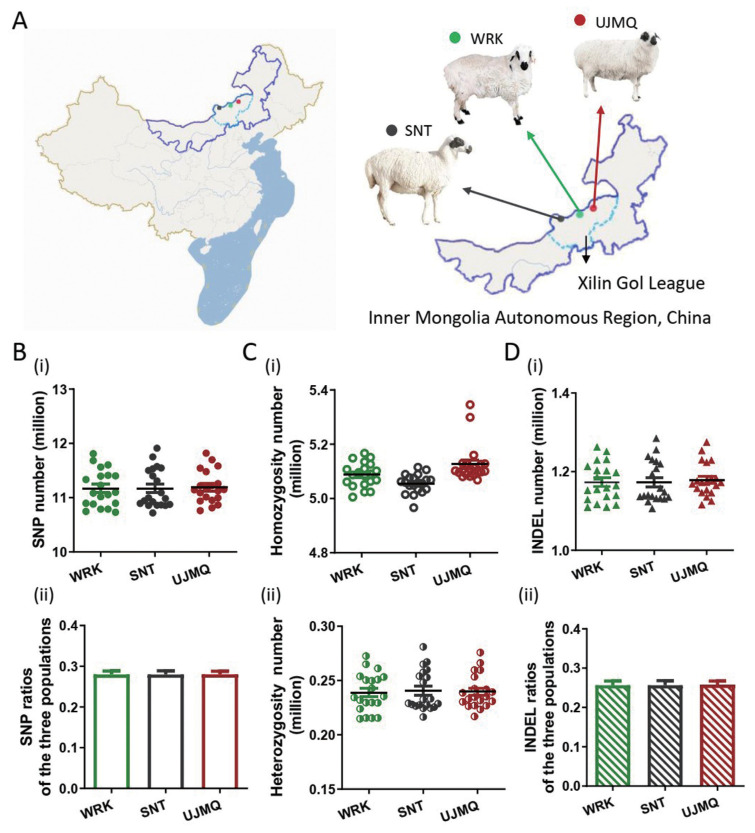
Experimental design and genetic variation. (A) Geographic distribution of three sheep breeds in this study. (B) The SNPs among three sheep breeds. (C) The homozygosity, and heterozygosity numbers. (D) The INDELs among WRK, SNT, and UJMQ. SNP, single nucleotide polymorphism; UJMQ, Ujimqin sheep; SNT, Sunit sheep; WRK, Wu Ranke sheep.

WRK, SNT, and UJMQ are local sheep breeds, which are mainly distributed in Abaga Banner, Sonid Left and Right Banners, East and West Ujimqin Banners, respectively. In our study, we sequenced 20 WRK, 20 SNT, and 20 UJMQ from local grazing breeding sheep populations in three regions of Inner Mongolia (Figure 1A). There was an average of 10× coverage per individual (~33.75 Gb of high-quality paired-end sequence data) after filtering and quality control, resulting in 13.50 billion mappable reads across 60 sheep.

We further identified about 45.9 million variants mainly including about 39.9 million SNPs and 4.6 million INDELs, which were not significantly different among three groups. The SNPs ranged from 10.7 to 11.8 million SNPs and the average SNPs per sample were 11.2 million (Figure 1B). The homozygosity and heterozygosity numbers were about 5.1 and 0.24 million, respectively (Figure 1C). In addition, the INDELs ranged from 11.2 to 12.7 million INDELs and the average INDELs per sample were 4.57 million (Figure 1D).

### Genome-wide association study among three sheep breeds

**Figure 2:**
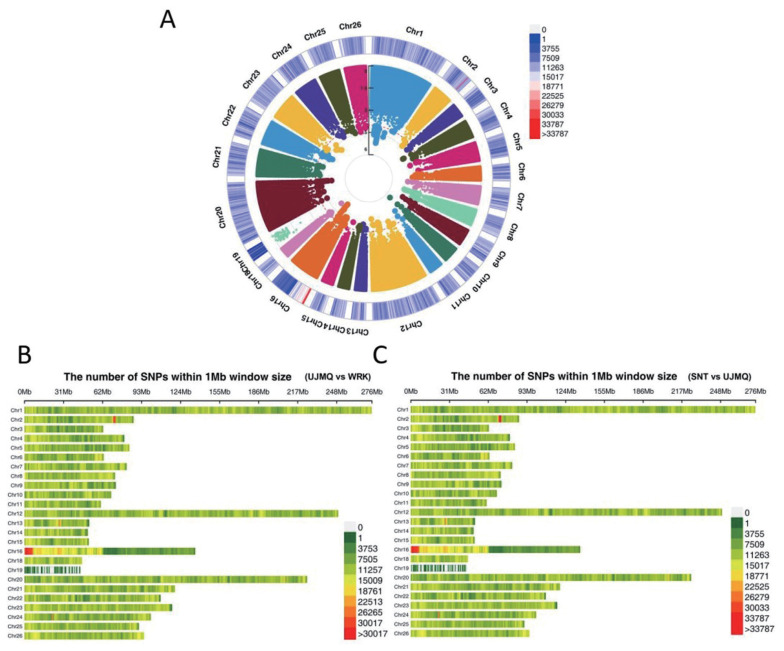
GWAS among three sheep breeds. (A) Manhattan plot of analysis results. (B) The number of SNPs between UJMQ and WRK. (C) The number of SNPs between SNT and UJMQ. GWAS, genome-wide association study; SNP, single nucleotide polymorphism; UJMQ, Ujimqin sheep; SNT, Sunit sheep; WRK, Wu Ranke sheep.

The GWAS was performed between three types of sheep breeds. The region of chromosome 16 (1 to 5 Mb) and chromosome 2 (65 to 67 Mb) displayed an ultra-high density of SNPs compared with other regions, which might play an important role on taxonomic and evolutionary implications for those breeds (Figure 2). A total of 468 and 779 significant (p<10^−5^) loci were identified from SNT versus UJMQ, and UJMQ versus WRK, respectively. However, only 3 loci were identified from SNT versus WRK, and those variations located in intron or intergenic regions which produced an insignificant effect on molecular phenotype. These results suggested that UJMQ were distinct from WRK and SNT, while SNT were very similar with WRK, similar with the maximum likelihood tree and PCA data.

### Population structure and domestication

**Figure 3:**
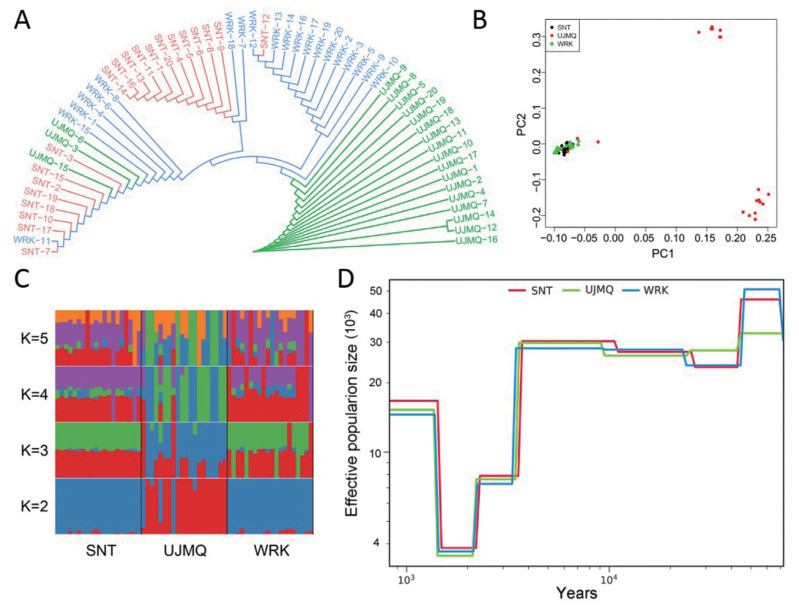
Population genetic structure and demographic history of three sheep populations. (A) Neighbor-joining phylogenetic tree of three sheep populations. (B) PCA plot of sheep populations. (C) Population genetic structure of 60 sheep, where number of ancestral clusters were set from K = 2 to 5. (D) Demographic history of sheep populations. PCA, principal component analysis.

To further investigate the relationships among three sheep breeds, we performed the maximum likelihood tree based on the pairwise genetic distances and PCA based on their genomic variants among all individuals, revealing that UJMQ clustered into subgroups that were distinct from WRK and SNT, while WRK and SNT were genetically indistinguishable at an overall genomic level (Figures 3A, 3B). This finding is similar with the considerable genetic difference among Mongolian sheep including UJMQ, Tan sheep, Small-tail Han sheep, Hu sheep, and Bayinbuluke sheep [3]. We further performed the population structure which estimates individual ancestry and admixture by assuming the number of ancestry K. With K = 2, UJMQ were separated from WRK and SNT, while WRK showed a mixture with SNT. With K increasing, a clear division was found between UJMQ and WRK or SNT. There were still no obvious division between WRK and SNT, in agreement with the PCA data (Figure 3C). We also explored the demographic history of our samples to differentiate whether domestication of Mongolian sheep including UJMQ, WRK, and SNT, was the result of one or multiple events (Figure 3D). The WRK populations have exhibited concordant demographic trajectories with SNT populations with three apparent contractions, followed by a subsequent expansion since 70 kya which is towards the end of the interglacial period (130 to 70 kya). UJMQ populations differentiated from WRK/SNT populations before the last glacial maximum (LGM) around 26 to 70 kya. Last glacial period (LGP) later, all populations showed similar trajectories. In detail, from a peak of about 45,000 to 50,000 during 45 to 70 kya, the population size of WRK and SNT populations dropped to about 23,000 between 45 to 26 kya during LGP, increased to about 28,000 at about 26.5 kya, and remained stable until 10 kya. Afterwards, SNT populations showed an increase and WRK populations remained stable until 3.5 kya. Differently, from a peak of about 33,000 at 45 kya, the population size of UJMQ dropped between 35-9.5 kya. All populations decreased the population size to about 8,000 between 4-3 kya, and further dropped to a small number between 2.5-2 kya and reached to 15,000–17,500 since about 1.5 kya. Next, we tested multiple demographic models using a diffusion approximation method for the allele frequency spectrum (*∂*a*∂*i). The demographic model also supported the aforementioned results, and the maximum log composite likelihood value of the optimal model were −1,157.57, −1,151.21, and −1,055.20 (Supplementary Figure S1).

Taken together, based on population genetic structure and demographic history analysis, we concluded that SNT and WRK populations were very similar, which were different from UJMQ populations.

### Selection for domestication

**Figure 4:**
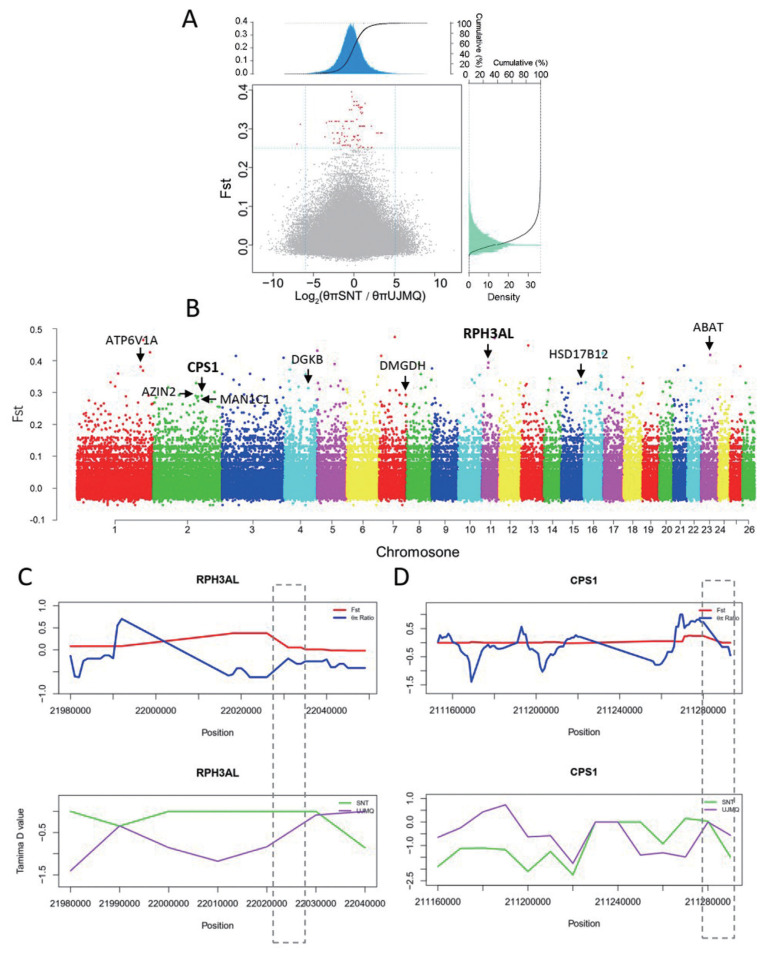
Genomic regions with strong selective sweep signals between SNT and UJMQ. (A) Distribution of –Log_2_(θ_π_ ratios) and *F*st values calculated with a sliding window approach (100 kb windows with 10 kb increments). (B) Genome-wide distribution of global *F*st. (C) –Log_2_(θ_π_ ratios) and *F*st values around the RPH3AL locus. Tajima’s D values around the RPH3AL locus. The red line and blue line indicate *F*st and θ_π_ ratios, respectively. (D) –Log_2_(θ_π_ ratios) and *F*st values around the CPS1 locus. Tajima’s D values around the CPS1 locus. The green line and purple line indicate UJMQ and WRK, respectively. UJMQ, Ujimqin sheep; SNT, Sunit sheep; WRK, Wu Ranke sheep.

**Figure 5:**
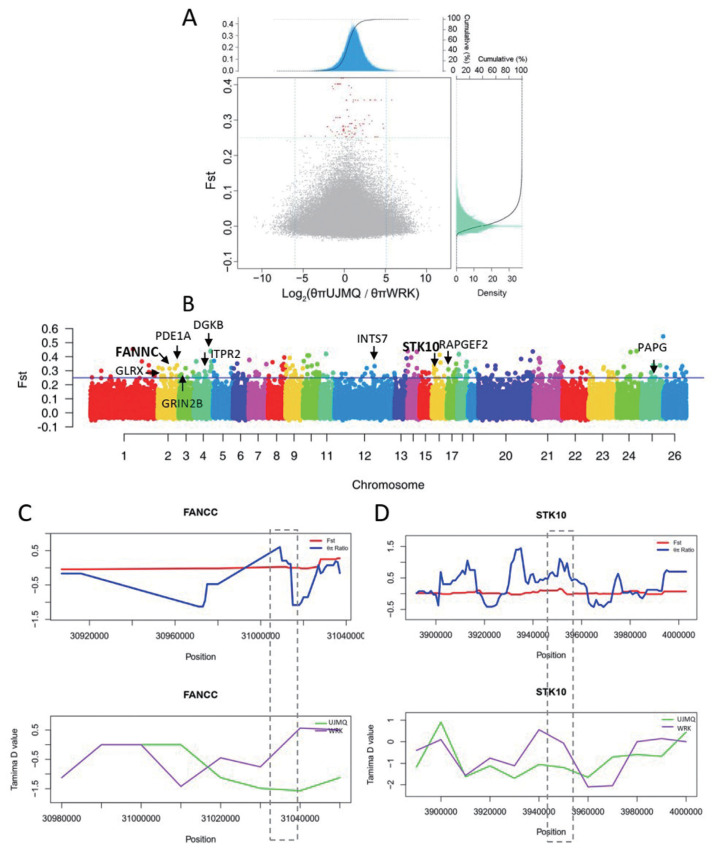
Genomic regions with strong selective sweep signals between UJMQ and WRK. (A) Distribution of –Log_2_(θ_π_ ratios) and *F*st values calculated with a sliding window approach (100 kb windows with 10 kb increments). (B) Genome-wide distribution of global *F*st. (C) –Log_2_(θ_π_ ratios) and *F*st values around the FANCC locus. Tajima’s D values around the FANCC locus. The red line and blue line indicate *F*st and θ_π_ ratios, respectively. (D) –Log_2_(θ_π_ ratios) and *F*st values around the STK10 locus. Tajima’s D values around the STK10 locus. The green line and purple line indicate UJMQ and WRK, respectively. UJMQ, Ujimqin sheep; WRK, Wu Ranke sheep.

To investigate the signature of selection for traits related to sheep domestication, we identified the genomic regions with a high *F*st and nucleotide diversity ratio (*θ*π) between the populations of UJMQ, WRK, and SNT. We measured allele frequency divergence as *F*st between species in 100 kb windows with 10 kb increments (Figure 4). Based on *F*st>0.25 and selecting the windows with t a relatively high or low diversity ratios, i.e., low diversity in the SNT population but high in the UJMQ population. Finally, we identified 292 putative selective sweeps located within 75 genes between UJMQ and WRK, and 262 putative selective sweeps located within 94 genes between SNT and UJMQ, which were under selection during domestication (Figures 4A, 5A). As WRK and SNT were very similar based on analysis mentioned above, only 18 significant loci and 2 genes between WRK and SNT (Supplementary Figure S2).

The GO terms and KEGG pathways were further analyzed (Supplementary Tables S1, S2). In the comparison of SNT to UJMQ, a total of 34 GO terms were significantly enriched, mainly including gamma-aminobutyric acid catabolic/metabolic process, response to cocaine/anesthetic/alkaloid, estrogen biosynthetic/metabolic process, transition metal ion homeostasis, regulation of exocytosis, and other metabolic process. A total of 3 KEGG pathways were significantly enriched and focused on metabolic pathways. From the highlighted GO and KEGG analysis, genes including 4-aminobutyrate aminotransferase (*ABAT*), ATPase H+ transporting V1 subunit A (*ATP6V1A*), hydroxysteroid 17-beta dehydrogenase 12 (*HSD17B12*), rabphilin 3A like (without C2 domains) (*RPH3AL*), carbamoyl-phosphate synthase 1 (*CPS1*), antizyme inhibitor 2 (*AZIN2*), dimethylglycine dehydrogenase (*DMGDH*), diacylglycerol kinase beta (*DGKB*), and mannosidase alpha class 1C member 1 (*MAN1C1*) were identified as being under positive selection in *F*st>0.25 (Figure 4B). Particularly, RPH3AL and CPS1 showed high *F*st and *θ*π value but lower Tajima’s D values compared to neighboring regions, indicating functional importance (Figures 4C, 4D).

In the comparison of UJMQ to WRK, a total of 86 GO terms were significantly enriched, mainly including metabolite biosynthetic process, response to cGMP/cAMP, and neuron development and differentiation. KEGG pathways including Long-term potentiation, phosphatidylinositol signaling system, calcium signaling pathway, and Alzheimer’s disease, were significantly enriched. From the highlighted GO and KEGG analysis (Supplementary Tables S3, S4), genes including FA complementation group C (*FANCC*), glutaredoxin (*GLRX*), integrator complex subunit 7 (*INTS7*), inositol 1,4,5-trisphosphate receptor type 2 (*ITPR2*), poly (ADP-ribose) glycohydrolase (*PARG*), phosphodiesterase 1A (*PDE1A*), Rap guanine nucleotide exchange factor 2 (*RAPGEF2*), serine/threonine kinase 10 (*STK10*), glutamate ionotropic receptor NMDA type subunit 2B (*GRIN2B*), and diacylglycerol kinase bet (*DGKB*) were identified as being under positive selection in *F*st>0.25 (Figure 5B). Importantly, SKT10 and FANNC showed high *F*st and *θ*π value, but lower Tajima’s D values compared to neighboring regions, suggesting that a strong selective sweep occurred in these genes (Figures 5C, 5D).

## DISCUSSION

The availability of whole-genome resequencing contributes to exploring genetic basis of animal domestication at the genomic level. Recently, population structure, domestication, and selection in wild and improved sheep with distinct phenotypes have caused more attention. The Mongolian sheep has been the most widely distributed in China, mainly including Inner Mongolia areas, eastern coastal regions, and the Central Plains, due to their great adaptability to the environment and Genghis Khan’s expedition in the Yuan dynasty [28]. Different Mongolian sheep breeds including WRK, SNT, UJMQ, and Hulunbuir sheep are living in different regions of Inner Mongolian Grassland, leading to their unique meat quality and flavors. However, few reports focused on comparison of these Mongolian sheep breeds at the genomic level. In previous report, Ganbold et al [29]. performed phylogenetic analysis among 88 Mongolian native sheep and other breeds located in different geographic regions in Eurasia and Africa by displacement loop region in mtDNA, which was an ingenious method to reveal the genetic differentiation and relationship. However, the amount of data limited the accuracy for the population structure and genetic differentiation. Whole genome sequencing can provide more reliable information for further excavation with public datasets. In this study, we performed whole-genome resequencing of 60 sheep from 3 Chinese indigenous Mongolian sheep breeds including UJMQ, SNT, and WRK. To our knowledge, this is the first whole-genome study to systematically illuminate genetic variation, population structure, domestication, and selection among Chinese indigenous Mongolian sheep breeds in different regions around Xilingol Grassland in China.

We focused on the relationships among three sheep breeds and found that SNT and WRK populations were very similar at the genomic level, suggesting that SNT and WRK might be from the same Mongolian sheep population in history and be distributed in west regions, and central-north areas of Xilingol Grassland in China, respectively. UJMQ living in northeast of Xilingol Grassland were a bit different from them in population structure and domestication. At the end of the interglacial period and during LGP (about 45 to 70 kya), UJMQ population size was lower than that in SNT and WRK. Subsequently, all these populations declined at about 45 kya. SNT and WRK showed a larger reduction than UJMQ and then slightly increased, leading to the similar population size among them until the LGM. The glaciations became less extensive and grassland expansion has occurred during LGM [6,30,31], which contributed to the expansion of grassland livestock. Consistently, SNT and WRK population sizes increased at the beginning of LGM (about 26.5 kya). After the LGP, all populations showed two apparent contradictions, one being 4 to 3 kya and the other being 2.5 to 2 kya. The phenomena may have resulted from Mongolian sheep spreading in the upper and middle reaches of the Yellow River about 3 to 5 kya and reaching the Qinghai-Tibetan and Yunnan-Kweichow plateaus about 2 to 2.6 kya from the Mongolian Plateau [5]. Later, a bottleneck with the lowest population size occurred at about 2.3 to 1.5 kya and the population size increased to about 17,000 at 1.5 kya.

To identify genomic regions that have been the selection target during domestication, we screened 94 genes between SNT and UJMQ, and 75 genes between UJMQ and WRK, based on *F*st>0.25. Between SNT and UJMQ, 94 genes were closely associated with metabolic pathways, implying the selection of these functionalities during domestication. Two loci, CPS1 and RPH3AL, exhibited strong signs of selective sweeps presumably related to domestication. CPS1, short for Carbamoyl-Phosphate Synthase 1, provides instructions for making the enzyme carbamoyl phosphate synthetase I. Sheep CPS1 was associated with amino acid metabolism including alanine, aspartate and glutamate metabolism, and arginine and proline metabolism. It has been reported CPS1 is a liver-specific enzyme converting ammonia to carbamoyl phosphate in the first step of the urea cycle [32,33]. RPH3AL that is a Rab effector was mainly related to exocytosis and regulation of secretion/exocytosis in sheep, which was consistent with previous studies in the mouse model [34]. To better understand the molecular characteristics of genes regulating some phenotypes is important. For instance, the polymorphism of melanocortin 1 receptor (*MC1R*) coding region in different coat colors in Mongolian goats have been investigated [35]. In the future, we can explore the polymorphism of some genes under positive selection to investigate deeply the molecular similarities and differences between UJMQ and SNT/WRK.

Xilingol region has different grassland types, such as, meadow steppes, typical steppes, and desert steppe, distributed in different areas [36]. Each steppe includes different construction plant species [9]. For instance, construction plant species in desert steppes includes *Allium polyrhizum* Turcz. ex Regel and several species of *Stipa. Stipa baicalensis* Roshev, *Bothriochloa ischaemum* L. Keng, *Cleistogenes mucronata* Keng, *L. angustum* (Trin.) Pilger, *Leymus chinensis* Tzvel., and *Folifolium sibiricum* are mainly in meadow steppes. Typical steppes mainly include *Cleistogenes squarrosa* (Trin.) Keng, *Agropyron cristatum* (L.) Gaertner, *Leymus chinensis* Tzvel., *A. intramongolica* HC Fu & ZY Zhu, *Artemisia frigida* Willd, *A. gmelinii* Webb ex Stechmann, and *Thymus mongolicus* Ronn [9]. Importantly, west parts of Xilingol League where SNT mainly are living belong to the desert steppe. Central-north areas in which WRK are mainly living belong to the typical steppe. While, northeast areas where UJMQ are distributed belong to the typical steppe and meadow steppe [36]. Thus, SNT, UJMQ, and WRK are distributed in regions with various grassland types and feed on different plants as forages, implying different gene profiles regulating metabolite biosynthetic process, which is consistent with our findings. The SNT and WRK varieties were genetically similar throughout the study despite the difference in the main habitats of desert steppes and typical steppes, respectively. In addition to different forages, these sheep are affected by a series of factors including climate, geography, and environment during evolution, which may lead to genetically very similar results between SNT and WRT varieties.

Taken together, according to the whole-genome resequencing, we found that WRK and SNT populations were very similar, which were different from UJMQ populations. Genes associated with regulation of secretion, metabolic pathways, and neuron development have undergone strong selection during domestication. These results will contribute to understanding Chinese indigenous Mongolian sheep breeds domestication and selection for complex traits and provide a valuable genomic resource for future studies of sheep and other domestic animal breeding.

## SUPPLEMENTARY MATERIAL

Supplementary Figure S1. Two-dimensional comparison for joint estimation between SNT and UJMQ (a), WRK and UJMQ (b), SNT and WRK (c) sheep breeds populations.

Supplementary Figure S2. Genome wide analysis of global Fst between WRK and SNT populations.

Supplementary Table S1. GO terms between SNT and UJMQ

Supplementary Table S2. KEGG between SNT and UJMQ

Supplementary Table S3. GO terms between UJMQ and WRK

Supplementary Table S4. KEGG between UJMQ and WRK
