# Supplementary material for: Whole-genome resequencing reveals domestication and signatures of selection in Ujimqin, Sunit, and Wu Ranke Mongolian sheep breeds
Source: Anim Biosci. 2022 Apr 29;35(9):1303–13. doi: 10.5713/ab.21.0569 (PMC9449395; doi:10.5713/ab.21.0569)
Supplement: Supplementary Figure S1. — Two-dimensional comparison for joint estimation between SNT and UJMQ (a), WRK and UJMQ (b), SNT and WRK (c) sheep breeds populations. [file ab-21-0569-suppl1.pdf]

Whole-genome resequencing reveals domestication and signatures of selection in Ujimqin, Sunit, and Wu Ranke Mongolian sheep breeds

Hanning *Wang*[1a], Liang *Zhong*[2a], Yanbing *Dong*[1], Lingbo *Meng*[1], Cheng *Ji*[1], Hui *Luo*[1], Mengrong *Fu*[1], Zhi *Qi*[1*], and Lan *Mi*[1*]

***Corresponding Author: Lan Mi**

E-mail: lanmi_90@126.com

Zhi Qi

E-mail: qizhi@imu.edu.cn

[1] State Key Laboratory of Reproductive Regulation and Breeding of Grassland Livestock, School of Life Sciences, Inner Mongolia University, Hohhot 020020, China

[2] Hebei Provincial Key Laboratory of Basic Medicine for Diabetes, The Shijiazhuang Second Hospital, Shijiazhuang 050051, China

[a] These authors contributed equally to this work.

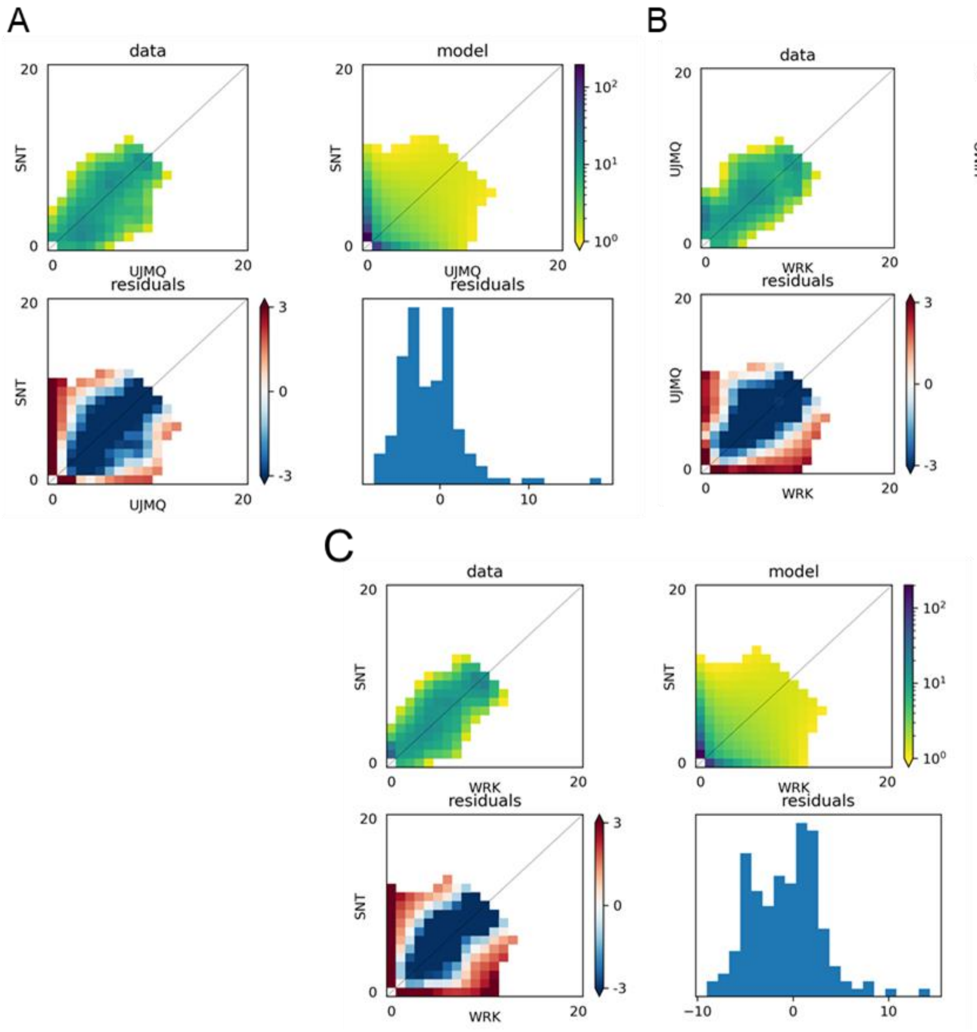

Figure S1. Two-dimensional comparison for joint estimation between SNT and UJMQ (**a**), WRK and UJMQ (**b**), SNT and WRK (**c**) sheep breeds populations. The two panels upside are marginal spectra for data and the maximum-likelihood model, respectively. The two panels downside show the residuals.

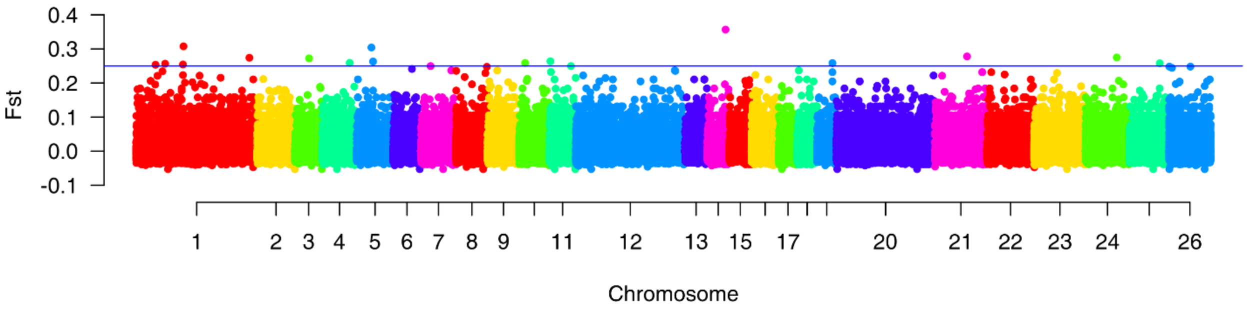

Figure S2. Genome-wide analysis of global *Fst* between WRK and SNT populations.
