# Supplementary material for: Whole-genome resequencing reveals domestication and signatures of selection in Ujimqin, Sunit, and Wu Ranke Mongolian sheep breeds
Source: Anim Biosci. 2022 Apr 29;35(9):1303–13. doi: 10.5713/ab.21.0569 (PMC9449395; doi:10.5713/ab.21.0569)
Supplement: Supplementary Table S1. — GO terms between SNT and UJMQ [file ab-21-0569-suppl3.pdf]

Whole-genome resequencing reveals domestication and signatures of selection in Ujimqin, Sunit, and Wu Ranke Mongolian sheep breeds

Hanning *Wang*[1a], Liang *Zhong*[2a], Yanbing *Dong*[1], Lingbo *Meng*[1], Cheng *Ji*[1], Hui *Luo*[1], Mengrong *Fu*[1], Zhi *Qi*[1*], and Lan *Mi*[1*]

***Corresponding Author: Lan Mi**

E-mail: lanmi_90@126.com

Zhi Qi

E-mail: qizhi@imu.edu.cn

[1] State Key Laboratory of Reproductive Regulation and Breeding of Grassland Livestock, School of Life Sciences, Inner Mongolia University, Hohhot 020020, China

[2] Hebei Provincial Key Laboratory of Basic Medicine for Diabetes, The Shijiazhuang Second Hospital, Shijiazhuang 050051, China

[a] These authors contributed equally to this work.

Table S1 GO terms between SNT and UJMQ

	P-value	Term	Symbols
1	0.001324	gamma-aminobutyric acid catabolic process	ABAT
2	0.002647	gamma-aminobutyric acid metabolic process	ABAT
3	0.003968	estrogen biosynthetic process	HSD17B12
4	0.005288	estrogen metabolic process	HSD17B12
5	0.005288	behavioral response to cocaine	ABAT
6	0.006606	response to cocaine	ABAT
7	0.009239	neurotransmitter catabolic process	ABAT
8	0.009239	response to anesthetic	ABAT
9	0.010553	cellular response to increased oxygen levels	ATP6V1A
10	0.010553	response to increased oxygen levels	ATP6V1A
11	0.013467	monocarboxylic acid metabolic process	ABAT;HSD17B12
12	0.014486	response to alkaloid	ABAT
13	0.017101	cellular iron ion homeostasis	ATP6V1A
14	0.021013	hormone biosynthetic process	HSD17B12
15	0.024911	cellular transition metal ion homeostasis	ATP6V1A
16	0.026208	energy coupled proton transmembrane transport, against electrochemical gradient	ATP6V1A
17	0.026208	ATP hydrolysis coupled proton transport	ATP6V1A
18	0.026208	iron ion homeostasis	ATP6V1A
19	0.026208	response to ammonium ion	ABAT
20	0.026208	ATP hydrolysis coupled transmembrane transport	ATP6V1A
21	0.026208	ATP hydrolysis coupled ion transmembrane transport	ATP6V1A
22	0.027503	monocarboxylic acid catabolic process	ABAT
23	0.028439	small molecule metabolic process	ABAT;ATP6V1A;HSD17B12
24	0.035244	transition metal ion homeostasis	ATP6V1A
25	0.03653	cellular response to oxygen levels	ATP6V1A
26	0.039096	response to xenobiotic stimulus	ABAT
27	0.039096	cellular hormone metabolic process	HSD17B12
28	0.041656	adult behavior	ABAT
29	0.044211	ATP hydrolysis coupled cation transmembrane transport	ATP6V1A
30	0.04617	carboxylic acid metabolic process	ABAT;HSD17B12
31	0.04676	regulation of exocytosis	RPH3AL
32	0.04676	neurotransmitter metabolic process	ABAT
33	0.049303	steroid biosynthetic process	HSD17B12
34	0.049642	oxoacid metabolic process	ABAT;HSD17B12
35	0.051578	organic acid metabolic process	ABAT;HSD17B12
36	0.053108	cellular amino acid catabolic process	ABAT
37	0.065698	fatty acid biosynthetic process	HSD17B12
38	0.065698	hormone metabolic process	HSD17B12

39	0.066238	regulation of biological quality	ABAT;ATP6V1A;HSD17B12
40	0.069448	response to oxygen levels	ATP6V1A
41	0.081856	regulation of neurotransmitter levels	ABAT
42	0.083089	proton transmembrane transport	ATP6V1A
43	0.086781	organic acid catabolic process	ABAT
44	0.086781	carboxylic acid catabolic process	ABAT
45	0.089235	exocytosis	RPH3AL
46	0.09046	steroid metabolic process	HSD17B12
47	0.10626	ATP metabolic process	ATP6V1A
48	0.10626	regulation of vesicle-mediated transport	RPH3AL
49	0.107466	monocarboxylic acid biosynthetic process	HSD17B12
50	0.111075	fatty acid metabolic process	HSD17B12
51	0.114672	cellular metal ion homeostasis	ATP6V1A
52	0.114672	purine ribonucleoside triphosphate metabolic process	ATP6V1A
53	0.117064	purine nucleoside triphosphate metabolic process	ATP6V1A
54	0.117064	ribonucleoside triphosphate metabolic process	ATP6V1A
55	0.11945	cellular amino acid metabolic process	ABAT
56	0.123019	purine nucleoside monophosphate metabolic process	ATP6V1A
57	0.123019	purine ribonucleoside monophosphate metabolic process	ATP6V1A
58	0.123019	regulation of hormone levels	HSD17B12
59	0.124206	behavior	ABAT
60	0.124206	small molecule catabolic process	ABAT
61	0.126576	nucleoside triphosphate metabolic process	ATP6V1A
62	0.127759	ribonucleoside monophosphate metabolic process	ATP6V1A
63	0.1313	cellular cation homeostasis	ATP6V1A
64	0.132477	nucleoside monophosphate metabolic process	ATP6V1A
65	0.133654	metal ion homeostasis	ATP6V1A
66	0.136002	cellular ion homeostasis	ATP6V1A
67	0.148825	carboxylic acid biosynthetic process	HSD17B12
68	0.149983	organic acid biosynthetic process	HSD17B12
69	0.152295	cation homeostasis	ATP6V1A
70	0.154601	inorganic ion homeostasis	ATP6V1A
71	0.155752	regulation of secretion by cell	RPH3AL
72	0.158259	biological regulation	ABAT;ATP6V1A;ADGRE5;RPH3AL;HSD17B12
73	0.161489	cellular chemical homeostasis	ATP6V1A
74	0.162632	monovalent inorganic cation transport	ATP6V1A
75	0.163774	purine ribonucleotide metabolic process	ATP6V1A

76	0.16833	ribonucleotide metabolic process	ATP6V1A
77	0.169466	purine nucleotide metabolic process	ATP6V1A
78	0.169466	response to organic cyclic compound	ABAT
79	0.169466	ribose phosphate metabolic process	ATP6V1A
80	0.172865	regulation of secretion	RPH3AL
81	0.175124	ion homeostasis	ATP6V1A
82	0.187461	purine-containing compound metabolic process	ATP6V1A
83	0.191909	lipid biosynthetic process	HSD17B12
84	0.194125	intracellular protein transport	RPH3AL
85	0.195231	response to organonitrogen compound	ABAT
86	0.19744	inorganic cation transmembrane transport	ATP6V1A
87	0.202939	nucleotide metabolic process	ATP6V1A
88	0.204035	response to drug	ABAT
89	0.206224	nucleoside phosphate metabolic process	ATP6V1A
90	0.209497	cellular homeostasis	ATP6V1A
91	0.218171	inorganic ion transmembrane transport	ATP6V1A
92	0.219249	secretion by cell	RPH3AL
93	0.219249	cation transmembrane transport	ATP6V1A
94	0.223552	response to nitrogen compound	ABAT
95	0.23634	nucleobase-containing small molecule metabolic process	ATP6V1A
96	0.239509	small molecule biosynthetic process	HSD17B12
97	0.248952	secretion	RPH3AL
98	0.251037	response to abiotic stimulus	ATP6V1A
99	0.251037	chemical homeostasis	ATP6V1A
100	0.259329	drug metabolic process	ATP6V1A
101	0.276695	ion transmembrane transport	ATP6V1A
102	0.279659	response to chemical	ABAT;ATP6V1A
103	0.279725	cell adhesion	ADGRE5
104	0.283747	translation	MRPL19
105	0.283747	cellular lipid metabolic process	HSD17B12
106	0.28475	biological adhesion	ADGRE5
107	0.288749	peptide biosynthetic process	MRPL19
108	0.297679	cation transport	ATP6V1A
109	0.308466	organophosphate metabolic process	ATP6V1A
110	0.310412	response to oxygen-containing compound	ABAT
111	0.315258	amide biosynthetic process	MRPL19
112	0.315258	organonitrogen compound catabolic process	ABAT
113	0.318152	peptide metabolic process	MRPL19
114	0.322953	carbohydrate derivative metabolic process	ATP6V1A
115	0.327725	intracellular transport	RPH3AL
116	0.343742	cellular protein localization	RPH3AL
117	0.345605	cellular macromolecule localization	RPH3AL

118	0.347196	cellular process	ABAT;ATP6V1A;ADGRE5;MRPL19;RPH3AL;HSD17B12
119	0.350244	vesicle-mediated transport	RPH3AL
120	0.363088	regulation of transport	RPH3AL
121	0.367623	cellular amide metabolic process	MRPL19
122	0.374823	transmembrane transport	ATP6V1A
123	0.376613	lipid metabolic process	HSD17B12
124	0.38814	homeostatic process	ATP6V1A
125	0.389898	establishment of localization in cell	RPH3AL
126	0.392061	organonitrogen compound metabolic process	ABAT;ATP6V1A;MRPL19
127	0.405528	ion transport	ATP6V1A
128	0.408955	protein transport	RPH3AL
129	0.416607	peptide transport	RPH3AL
130	0.423339	amide transport	RPH3AL
131	0.424176	establishment of protein localization	RPH3AL
132	0.425847	oxidation-reduction process	HSD17B12
133	0.427103	transport	ATP6V1A;RPH3AL
134	0.437426	primary metabolic process	ABAT;ATP6V1A;MRPL19;HSD17B12
135	0.440943	establishment of localization	ATP6V1A;RPH3AL
136	0.443962	cell surface receptor signaling pathway	ADGRE5
137	0.456671	cellular metabolic process	ABAT;ATP6V1A;MRPL19;HSD17B12
138	0.45916	response to stimulus	ABAT;ATP6V1A;ADGRE5
139	0.471019	cellular catabolic process	ABAT
140	0.476451	organic substance catabolic process	ABAT
141	0.491715	nitrogen compound transport	RPH3AL
142	0.498462	cellular localization	RPH3AL
143	0.499245	organic substance metabolic process	ABAT;ATP6V1A;MRPL19;HSD17B12
144	0.502178	regulation of localization	RPH3AL
145	0.523996	protein localization	RPH3AL
146	0.528262	catabolic process	ABAT
147	0.52897	organonitrogen compound biosynthetic process	MRPL19
148	0.529883	cellular biosynthetic process	MRPL19;HSD17B12
149	0.534882	organic cyclic compound metabolic process	ATP6V1A;HSD17B12
150	0.535302	response to organic substance	ABAT
151	0.546296	organic substance biosynthetic process	MRPL19;HSD17B12
152	0.54912	organic substance transport	RPH3AL
153	0.553197	cellular response to chemical stimulus	ATP6V1A
154	0.559054	biosynthetic process	MRPL19;HSD17B12
155	0.571171	localization	ATP6V1A;RPH3AL
156	0.577024	macromolecule localization	RPH3AL
157	0.609752	metabolic process	ABAT;ATP6V1A;MRPL19;HSD17B12
158	0.611654	cellular response to stimulus	ATP6V1A;ADGRE5

159	0.613031	cellular nitrogen compound metabolic process	ATP6V1A;MRPL19
160	0.643932	phosphate-containing compound metabolic process	ATP6V1A
161	0.64561	phosphorus metabolic process	ATP6V1A
162	0.672967	nitrogen compound metabolic process	ABAT;ATP6V1A;MRPL19
163	0.674183	organic cyclic compound biosynthetic process	HSD17B12
164	0.743578	cellular macromolecule biosynthetic process	MRPL19
165	0.764563	macromolecule biosynthetic process	MRPL19
166	0.766146	cellular nitrogen compound biosynthetic process	MRPL19
167	0.797056	gene expression	MRPL19
168	0.799151	signal transduction	ADGRE5
169	0.818175	signaling	ADGRE5
170	0.824483	cell communication	ADGRE5
171	0.825414	nucleobase-containing compound metabolic process	ATP6V1A
172	0.838883	heterocycle metabolic process	ATP6V1A
173	0.843458	cellular aromatic compound metabolic process	ATP6V1A
174	0.846262	cellular protein metabolic process	MRPL19
175	0.847017	regulation of cellular process	ADGRE5;RPH3AL
176	0.879982	multicellular organismal process	ABAT
177	0.88575	protein metabolic process	MRPL19
178	0.892937	regulation of biological process	ADGRE5;RPH3AL
179	0.947792	cellular macromolecule metabolic process	MRPL19
180	0.975561	macromolecule metabolic process	MRPL19
181	0.001538	4-aminobutyrate transaminase activity	ABAT
182	0.001538	4-aminobutyrate:2-oxoglutarate transaminase activity	ABAT
183	0.001538	(S)-3-amino-2-methylpropionate transaminase activity	ABAT
184	0.001538	3-oxo-arachidoyl-CoA reductase activity	HSD17B12
185	0.001538	3-oxo-behenoyl-CoA reductase activity	HSD17B12
186	0.001538	3-oxo-lignoceroyl-CoA reductase activity	HSD17B12
187	0.001538	3-oxo-cerotoyl-CoA reductase activity	HSD17B12
188	0.007671	estradiol 17-beta-dehydrogenase activity	HSD17B12
189	0.015292	steroid dehydrogenase activity, acting on the CH-OH group of donors, NAD or NADP as acceptor	HSD17B12
190	0.016811	transaminase activity	ABAT
191	0.016811	steroid dehydrogenase activity	HSD17B12

192	0.019842	transferase activity, transferring nitrogenous groups	ABAT
193	0.027385	Rab GTPase binding	RPH3AL
194	0.029576	calcium ion binding	ADGRE5;EFCAB3
195	0.043811	pyridoxal phosphate binding	ABAT
196	0.043811	vitamin B6 binding	ABAT
197	0.052672	iron-sulfur cluster binding	ABAT
198	0.052672	metal cluster binding	ABAT
199	0.052746	ion binding	ABAT;ATP6V1A;ADGRE5;EFCAB3;RPH3AL
200	0.056755	metal ion binding	ABAT;ADGRE5;EFCAB3;RPH3AL
201	0.058185	cation binding	ABAT;ADGRE5;EFCAB3;RPH3AL
202	0.067289	Ras GTPase binding	RPH3AL
203	0.067289	small GTPase binding	RPH3AL
204	0.073083	oxidoreductase activity, acting on the CH-OH group of donors, NAD or NADP as acceptor	HSD17B12
205	0.081718	oxidoreductase activity, acting on CH-OH group of donors	HSD17B12
206	0.094544	vitamin binding	ABAT
207	0.104417	GTPase binding	RPH3AL
208	0.147209	drug binding	ABAT;ATP6V1A
209	0.160458	coenzyme binding	ABAT
210	0.257824	cofactor binding	ABAT
211	0.270931	protein homodimerization activity	ABAT
212	0.311136	small molecule binding	ABAT;ATP6V1A
213	0.32183	anion binding	ABAT;ATP6V1A
214	0.365863	oxidoreductase activity	HSD17B12
215	0.37632	protein dimerization activity	ABAT
216	0.430337	enzyme binding	RPH3AL
217	0.451197	identical protein binding	ABAT
218	0.462303	ATP binding	ATP6V1A
219	0.475923	adenyl ribonucleotide binding	ATP6V1A
220	0.476821	adenyl nucleotide binding	ATP6V1A
221	0.530029	transferase activity	ABAT
222	0.559576	purine ribonucleoside triphosphate binding	ATP6V1A
223	0.576398	purine ribonucleotide binding	ATP6V1A
224	0.57715	purine nucleotide binding	ATP6V1A
225	0.5779	ribonucleotide binding	ATP6V1A
226	0.597024	binding	ABAT;ATP6V1A;ADGRE5;EFCAB3;RPH3AL
227	0.633129	carbohydrate derivative binding	ATP6V1A
228	0.635012	heterocyclic compound binding	ABAT;ATP6V1A
229	0.642373	nucleotide binding	ATP6V1A
230	0.642373	nucleoside phosphate binding	ATP6V1A
231	0.643237	organic cyclic compound binding	ABAT;ATP6V1A

232	0.70169	protein binding	ABAT;RPH3AL
233	0.712221	catalytic activity	ABAT;HSD17B12
234	0.001054	4-aminobutyrate transaminase complex	ABAT
235	0.006312	proton-transporting V-type ATPase, V1 domain	ATP6V1A
236	0.010502	proton-transporting two-sector ATPase complex, catalytic domain	ATP6V1A
237	0.01444	mitochondrial matrix	ABAT;MRPL19
238	0.01572	proton-transporting V-type ATPase complex	ATP6V1A
239	0.020915	microvillus	ATP6V1A
240	0.028364	cytoplasmic part	ABAT;ATP6V1A;MRPL19;RPH3AL;HSD17B12
241	0.040459	proton-transporting two-sector ATPase complex	ATP6V1A
242	0.044534	organellar large ribosomal subunit	MRPL19
243	0.044534	mitochondrial large ribosomal subunit	MRPL19
244	0.048595	large ribosomal subunit	MRPL19
245	0.048595	actin-based cell projection	ATP6V1A
246	0.05062	transport vesicle membrane	RPH3AL
247	0.066697	apical plasma membrane	ATP6V1A
248	0.075516	mitochondrial part	ABAT;MRPL19
249	0.075644	organellar ribosome	MRPL19
250	0.075644	mitochondrial ribosome	MRPL19
251	0.080585	apical part of cell	ATP6V1A
252	0.085505	transport vesicle	RPH3AL
253	0.088447	ribosomal subunit	MRPL19
254	0.101451	cytoplasm	ABAT;ATP6V1A;MRPL19;RPH3AL;HSD17B12
255	0.105939	ribosome	MRPL19
256	0.127433	protein-containing complex	ABAT;ATP6V1A;MRPL19
257	0.133614	mitochondrion	ABAT;MRPL19
258	0.140082	membrane	ATP6V1A;MRPL19;RPH3AL;HSD17B12
259	0.140117	cytoplasmic vesicle membrane	RPH3AL
260	0.141985	vesicle membrane	RPH3AL
261	0.14679	intracellular organelle part	ABAT;MRPL19;RPH3AL;HSD17B12
262	0.164428	organelle part	ABAT;MRPL19;RPH3AL;HSD17B12
263	0.165058	mitochondrial protein complex	MRPL19
264	0.167793	transferase complex	ABAT
265	0.227437	organelle membrane	MRPL19;RPH3AL
266	0.227893	cytoplasmic vesicle part	RPH3AL
267	0.246575	plasma membrane region	ATP6V1A
268	0.248255	ribonucleoprotein complex	MRPL19
269	0.254233	membrane-enclosed lumen	ABAT;MRPL19
270	0.254233	organelle lumen	ABAT;MRPL19
271	0.254233	intracellular organelle lumen	ABAT;MRPL19
272	0.258273	endoplasmic reticulum membrane	HSD17B12

273	0.259104	endoplasmic reticulum subcompartment	HSD17B12
274	0.263243	mitochondrial inner membrane	MRPL19
275	0.264068	nuclear outer membrane-endoplasmic reticulum membrane network	HSD17B12
276	0.275549	organelle inner membrane	MRPL19
277	0.286886	endoplasmic reticulum part	HSD17B12
278	0.29027	endomembrane system	RPH3AL;HSD17B12
279	0.297017	intracellular	ABAT;ATP6V1A;MRPL19;RPH3AL;HSD17B12
280	0.297017	intracellular part	ABAT;ATP6V1A;MRPL19;RPH3AL;HSD17B12
281	0.302835	membrane protein complex	ATP6V1A
282	0.320822	mitochondrial membrane	MRPL19
283	0.331588	mitochondrial envelope	MRPL19
284	0.331588	catalytic complex	ABAT
285	0.33616	whole membrane	RPH3AL
286	0.347203	membrane-bounded organelle	ABAT;MRPL19;RPH3AL;HSD17B12
287	0.363069	organelle subcompartment	HSD17B12
288	0.369658	plasma membrane bounded cell projection	ATP6V1A
289	0.381955	plasma membrane part	ATP6V1A
290	0.386962	cell projection	ATP6V1A
291	0.389098	endoplasmic reticulum	HSD17B12
292	0.40109	organelle envelope	MRPL19
293	0.40109	envelope	MRPL19
294	0.416329	bounding membrane of organelle	RPH3AL
295	0.417698	cytoplasmic vesicle	RPH3AL
296	0.417698	intracellular vesicle	RPH3AL
297	0.435273	vesicle	RPH3AL
298	0.473813	intracellular organelle	ABAT;MRPL19;RPH3AL;HSD17B12
299	0.484874	cell part	ABAT;ATP6V1A;MRPL19;RPH3AL;HSD17B12
300	0.495606	cell	ABAT;ATP6V1A;MRPL19;RPH3AL;HSD17B12
301	0.502257	organelle	ABAT;MRPL19;RPH3AL;HSD17B12
302	0.5326	cytosol	ATP6V1A
303	0.552015	membrane part	ATP6V1A;HSD17B12
304	0.604898	intracellular membrane-bounded organelle	ABAT;MRPL19;HSD17B12
305	0.633825	plasma membrane	ATP6V1A
306	0.645476	cell periphery	ATP6V1A
307	0.72242	non-membrane-bounded organelle	MRPL19
308	0.72242	intracellular non-membrane-bounded organelle	MRPL19
309	0.761944	integral component of membrane	HSD17B12
310	0.774067	intrinsic component of membrane	HSD17B12
