# Supplementary material for: Whole-genome resequencing reveals domestication and signatures of selection in Ujimqin, Sunit, and Wu Ranke Mongolian sheep breeds
Source: Anim Biosci. 2022 Apr 29;35(9):1303–13. doi: 10.5713/ab.21.0569 (PMC9449395; doi:10.5713/ab.21.0569)
Supplement: Supplementary Table S2. — KEGG between SNT and UJMQ [file ab-21-0569-suppl4.pdf]

Whole-genome resequencing reveals domestication and signatures of selection in Ujimqin, Sunit, and Wu Ranke Mongolian sheep breeds

Hanning *Wang*[1a], Liang *Zhong*[2a], Yanbing *Dong*[1], Lingbo *Meng*[1], Cheng *Ji*[1], Hui *Luo*[1], Mengrong *Fu*[1], Zhi *Qi*[1*], and Lan *Mi*[1*]

***Corresponding Author: Lan Mi**

E-mail: lanmi_90@126.com

Zhi Qi

E-mail: qizhi@imu.edu.cn

[1] State Key Laboratory of Reproductive Regulation and Breeding of Grassland Livestock, School of Life Sciences, Inner Mongolia University, Hohhot 020020, China

[2] Hebei Provincial Key Laboratory of Basic Medicine for Diabetes, The Shijiazhuang Second Hospital, Shijiazhuang 050051, China

[a] These authors contributed equally to this work.

Table S2 KEGG between SNT and UJMQ

	P-value	Term	Symbols
1	0.004536	Alanine, aspartate and glutamate metabolism	ABAT;CPS1
2	0.01322	Arginine and proline metabolism	AZIN2;CPS1
3	0.022065	Metabolic pathways	ABAT;ATP6V1A;DMGDH;AZIN2;CPS1;DGKB;MAN1C1;HSD17B12
4	0.057525	Vascular smooth muscle contraction	AVPR1B;ADCY2
5	0.063867	Axon guidance	PLXNA2;RGS3
6	0.066118	Dorso-ventral axis formation	ETS1
7	0.069313	Biosynthesis of unsaturated fatty acids	HSD17B12
8	0.07567	beta-Alanine metabolism	ABAT
9	0.078834	Nitrogen metabolism	CPS1
10	0.08513	Collecting duct acid secretion	ATP6V1A
11	0.091384	Butanoate metabolism	ABAT
12	0.103773	Propanoate metabolism	ABAT
13	0.10849	Protein processing in endoplasmic reticulum	MBTPS1;MAN1C1
14	0.116	Glycine, serine and threonine metabolism	DMGDH
15	0.122969	Calcium signaling pathway	AVPR1B;ADCY2
16	0.125067	Steroid hormone biosynthesis	HSD17B12
17	0.125067	Aminoacyl-tRNA biosynthesis	NARS2
18	0.139982	Valine, leucine and isoleucine degradation	ABAT
19	0.139982	Lysine degradation	SETD7
20	0.142936	N-Glycan biosynthesis	MAN1C1
21	0.157561	Glycerolipid metabolism	DGKB
22	0.16909	Cytosolic DNA-sensing pathway	RIPK1
23	0.197261	p53 signaling pathway	CHEK2
24	0.208274	Bile secretion	ADCY2
25	0.213726	RIG-I-like receptor signaling pathway	RIPK1
26	0.216438	Gastric acid secretion	ADCY2
27	0.232529	Salivary secretion	ADCY2
28	0.232529	Phosphatidylinositol signaling system	DGKB
29	0.243081	Glycerophospholipid metabolism	DGKB
30	0.245697	Dilated cardiomyopathy	ADCY2
31	0.248305	Apoptosis	RIPK1
32	0.253495	Progesterone-mediated oocyte maturation	ADCY2
33	0.263773	Rheumatoid arthritis	ATP6V1A

34	0.266321	Gap junction	ADCY2
35	0.278938	Melanogenesis	ADCY2
36	0.283927	Pancreatic secretion	ADCY2
37	0.283927	GnRH signaling pathway	ADCY2
38	0.293806	Toll-like receptor signaling pathway	RIPK1
39	0.33899	Oocyte meiosis	ADCY2
40	0.359422	Cell cycle	CHEK2
41	0.374897	Hepatitis C	RIPK1
42	0.394271	Oxidative phosphorylation	ATP6V1A
43	0.402698	Phagosome	ATP6V1A
44	0.402698	Wnt signaling pathway	DAAM2
45	0.406869	RNA transport	NUP107
46	0.425301	Purine metabolism	ADCY2
47	0.460544	Chemokine signaling pathway	ADCY2
48	0.608066	Neuroactive ligand-receptor interaction	AVPR1B
