# Supplementary material for: Whole-genome resequencing reveals domestication and signatures of selection in Ujimqin, Sunit, and Wu Ranke Mongolian sheep breeds
Source: Anim Biosci. 2022 Apr 29;35(9):1303–13. doi: 10.5713/ab.21.0569 (PMC9449395; doi:10.5713/ab.21.0569)
Supplement: Supplementary Table S3. — GO terms between UJMQ and WRK [file ab-21-0569-suppl5.pdf]

Whole-genome resequencing reveals domestication and signatures of selection in Ujimqin, Sunit, and Wu Ranke Mongolian sheep breeds

Hanning *Wang*[1a], Liang *Zhong*[2a], Yanbing *Dong*[1], Lingbo *Meng*[1], Cheng *Ji*[1], Hui *Luo*[1], Mengrong *Fu*[1], Zhi *Qi*[1*], and Lan *Mi*[1*]

***Corresponding Author: Lan Mi**

E-mail: lanmi_90@126.com

Zhi Qi

E-mail: qizhi@imu.edu.cn

[1] State Key Laboratory of Reproductive Regulation and Breeding of Grassland Livestock, School of Life Sciences, Inner Mongolia University, Hohhot 020020, China

[2] Hebei Provincial Key Laboratory of Basic Medicine for Diabetes, The Shijiazhuang Second Hospital, Shijiazhuang 050051, China

[a] These authors contributed equally to this work.

Table S3 GO terms between UJMQ and WRK

	P-value	Term	Symbols
1	0.001766	brain-derived neurotrophic factor receptor signaling pathway	RAPGEF2
2	0.001766	negative regulation of melanin biosynthetic process	RAPGEF2
3	0.001766	dendritic cell apoptotic process	RAPGEF2
4	0.001766	negative regulation of secondary metabolite biosynthetic process	RAPGEF2
5	0.001766	regulation of dendritic cell apoptotic process	RAPGEF2
6	0.001766	positive regulation of dendritic cell apoptotic process	RAPGEF2
7	0.001766	positive regulation of neuron migration	RAPGEF2
8	0.003529	regulation of melanin biosynthetic process	RAPGEF2
9	0.003529	response to cGMP	RAPGEF2
10	0.003529	cellular response to cGMP	RAPGEF2
11	0.003529	regulation of secondary metabolite biosynthetic process	RAPGEF2
12	0.003583	cellular response to DNA damage stimulus	PARG;FANCC;INTS7
13	0.005289	nerve growth factor signaling pathway	RAPGEF2
14	0.005289	negative regulation of dendrite morphogenesis	RAPGEF2
15	0.005289	ATP generation from poly-ADP-D-ribose	PARG
16	0.005289	negative regulation of dendrite development	RAPGEF2
17	0.005289	positive regulation of cAMP-dependent protein kinase activity	RAPGEF2
18	0.005289	positive regulation of vasculogenesis	RAPGEF2
19	0.006204	second-messenger-mediated signaling	RAPGEF2;ITPR2
20	0.007046	regulation of secondary metabolic process	RAPGEF2
21	0.007046	regulation of vasculogenesis	RAPGEF2
22	0.007046	regulation of neuron migration	RAPGEF2
23	0.008801	forebrain neuron development	RAPGEF2
24	0.008801	Rap protein signal transduction	RAPGEF2
25	0.008801	positive regulation of cAMP-mediated signaling	RAPGEF2
26	0.008801	adrenergic receptor signaling pathway	RAPGEF2
27	0.008801	adenylate cyclase-activating adrenergic receptor signaling pathway	RAPGEF2
28	0.010553	ventricular system development	RAPGEF2
29	0.010553	positive regulation of leukocyte apoptotic process	RAPGEF2

30	0.010553	regulation of cAMP-dependent protein kinase activity	RAPGEF2
31	0.012302	forebrain neuron differentiation	RAPGEF2
32	0.012302	interstrand cross-link repair	FANCC
33	0.012302	neurotrophin signaling pathway	RAPGEF2
34	0.012302	response to nerve growth factor	RAPGEF2
35	0.012302	cellular response to nerve growth factor stimulus	RAPGEF2
36	0.014529	cellular response to stimulus	RAPGEF2;PARG;FANCC;ITPR2;PDE1A;INTS7
37	0.015793	melanin metabolic process	RAPGEF2
38	0.015793	forebrain generation of neurons	RAPGEF2
39	0.015793	melanin biosynthetic process	RAPGEF2
40	0.015793	cellular response to cAMP	RAPGEF2
41	0.017534	central nervous system neuron development	RAPGEF2
42	0.017534	regulation of dendrite morphogenesis	RAPGEF2
43	0.019272	regulation of cAMP-mediated signaling	RAPGEF2
44	0.019272	secondary metabolite biosynthetic process	RAPGEF2
45	0.019272	response to cAMP	RAPGEF2
46	0.021008	vasculogenesis	RAPGEF2
47	0.021008	negative regulation of cell morphogenesis involved in differentiation	RAPGEF2
48	0.021008	establishment of endothelial barrier	RAPGEF2
49	0.024472	regulation of dendrite development	RAPGEF2
50	0.024472	cellular response to ionizing radiation	INTS7
51	0.02589	cellular response to stress	PARG;FANCC;INTS7
52	0.026199	endothelial cell development	RAPGEF2
53	0.026199	response to organophosphorus	RAPGEF2
54	0.026199	inositol phosphate-mediated signaling	ITPR2
55	0.026199	regulation of lymphocyte migration	STK10
56	0.027924	negative regulation of neuron projection development	RAPGEF2
57	0.027924	regulation of leukocyte apoptotic process	RAPGEF2
58	0.029647	secondary metabolic process	RAPGEF2
59	0.029647	dendrite morphogenesis	RAPGEF2
60	0.030871	regulation of cell migration	RAPGEF2;STK10
61	0.031367	response to purine-containing compound	RAPGEF2
62	0.033084	DNA damage checkpoint	INTS7
63	0.033084	endothelial cell differentiation	RAPGEF2
64	0.033084	phenol-containing compound biosynthetic process	RAPGEF2
65	0.033084	leukocyte apoptotic process	RAPGEF2
66	0.033084	lymphocyte migration	STK10
67	0.033764	regulation of cell motility	RAPGEF2;STK10

68	0.034798	DNA integrity checkpoint	INTS7
69	0.034798	regulation of cell junction assembly	RAPGEF2
70	0.036383	regulation of cellular component movement	RAPGEF2;STK10
71	0.03651	positive regulation of neuron projection development	RAPGEF2
72	0.03651	central nervous system neuron differentiation	RAPGEF2
73	0.03651	pigment biosynthetic process	RAPGEF2
74	0.038219	protein autophosphorylation	STK10
75	0.039925	endothelium development	RAPGEF2
76	0.039925	dendrite development	RAPGEF2
77	0.039925	pigment metabolic process	RAPGEF2
78	0.039925	negative regulation of neuron differentiation	RAPGEF2
79	0.040261	regulation of locomotion	RAPGEF2;STK10
80	0.041629	negative regulation of cell projection organization	RAPGEF2
81	0.04333	positive regulation of protein binding	RAPGEF2
82	0.045029	adenylate cyclase-activating G protein-coupled receptor signaling pathway	RAPGEF2
83	0.045029	cell redox homeostasis	GLRX
84	0.046724	neuron migration	RAPGEF2
85	0.048418	neuropeptide signaling pathway	RAPGEF2
86	0.048418	phenol-containing compound metabolic process	RAPGEF2
87	0.050702	response to stimulus	RAPGEF2;PARG;FANCC;ITPR2;PDE1A;INTS7
88	0.051796	positive regulation of neuron differentiation	RAPGEF2
89	0.051796	positive regulation of ERK1 and ERK2 cascade	RAPGEF2
90	0.053482	response to ionizing radiation	INTS7
91	0.056844	regulation of cell morphogenesis involved in differentiation	RAPGEF2
92	0.058522	cAMP-mediated signaling	RAPGEF2
93	0.058522	positive regulation of cell projection organization	RAPGEF2
94	0.060197	cellular response to radiation	INTS7
95	0.061869	cell cycle checkpoint	INTS7
96	0.061869	adenylate cyclase-modulating G protein-coupled receptor signaling pathway	RAPGEF2
97	0.061869	cyclic-nucleotide-mediated signaling	RAPGEF2
98	0.061869	negative regulation of neurogenesis	RAPGEF2

99	0.063539	G protein-coupled receptor signaling pathway, coupled to cyclic nucleotide second messenger	RAPGEF2
100	0.063539	cell junction assembly	RAPGEF2
101	0.063539	positive regulation of vasculature development	RAPGEF2
102	0.06475	cell migration	RAPGEF2;STK10
103	0.065206	regulation of leukocyte migration	STK10
104	0.06687	negative regulation of nervous system development	RAPGEF2
105	0.071848	epithelial cell development	RAPGEF2
106	0.071848	forebrain development	RAPGEF2
107	0.075153	cell junction organization	RAPGEF2
108	0.076802	negative regulation of cell development	RAPGEF2
109	0.076802	regulation of ERK1 and ERK2 cascade	RAPGEF2
110	0.077555	cell motility	RAPGEF2;STK10
111	0.077555	localization of cell	RAPGEF2;STK10
112	0.078449	positive regulation of neurogenesis	RAPGEF2
113	0.078449	positive regulation of binding	RAPGEF2
114	0.080092	ERK1 and ERK2 cascade	RAPGEF2
115	0.081734	regulation of protein binding	RAPGEF2
116	0.083372	regulation of neuron projection development	RAPGEF2
117	0.091527	organic hydroxy compound biosynthetic process	RAPGEF2
118	0.093151	cellular response to abiotic stimulus	INTS7
119	0.093151	cellular response to environmental stimulus	INTS7
120	0.09639	positive regulation of nervous system development	RAPGEF2
121	0.09639	positive regulation of protein serine/threonine kinase activity	RAPGEF2
122	0.096603	regulation of cellular process	RAPGEF2;ITPR2;PDE1A;GLRX;STK10;INTS7
123	0.098006	Ras protein signal transduction	RAPGEF2
124	0.102838	electron transport chain	GLRX
125	0.103228	movement of cell or subcellular component	RAPGEF2;STK10
126	0.105474	locomotion	RAPGEF2;STK10
127	0.106046	positive regulation of GTPase activity	RAPGEF2
128	0.106046	regulation of neuron differentiation	RAPGEF2
129	0.107647	small GTPase mediated signal transduction	RAPGEF2
130	0.114025	cell morphogenesis involved in neuron differentiation	RAPGEF2

131	0.115613	positive regulation of cell development	RAPGEF2
132	0.115613	regulation of vasculature development	RAPGEF2
133	0.117198	leukocyte migration	STK10
134	0.118889	phosphate-containing compound metabolic process	RAPGEF2;PARG;STK10
135	0.120067	phosphorus metabolic process	RAPGEF2;PARG;STK10
136	0.12194	neuron projection morphogenesis	RAPGEF2
137	0.123516	brain development	RAPGEF2
138	0.123516	regulation of GTPase activity	RAPGEF2
139	0.125089	regulation of binding	RAPGEF2
140	0.128228	regulation of cell morphogenesis	RAPGEF2
141	0.131358	cell projection morphogenesis	RAPGEF2
142	0.131358	plasma membrane bounded cell projection morphogenesis	RAPGEF2
143	0.13295	protein phosphorylation	RAPGEF2;STK10
144	0.133555	cell cycle	STK10;INTS7
145	0.134477	head development	RAPGEF2
146	0.137586	cell part morphogenesis	RAPGEF2
147	0.137586	positive regulation of protein kinase activity	RAPGEF2
148	0.139138	ATP metabolic process	PARG
149	0.143776	transmembrane receptor protein tyrosine kinase signaling pathway	RAPGEF2
150	0.146856	regulation of protein serine/threonine kinase activity	RAPGEF2
151	0.147376	regulation of biological process	RAPGEF2;ITPR2;PDE1A;GLRX;STK10;INTS7
152	0.148393	regulation of plasma membrane bounded cell projection organization	RAPGEF2
153	0.149927	cell morphogenesis involved in differentiation	RAPGEF2
154	0.149927	purine ribonucleoside triphosphate metabolic process	PARG
155	0.149927	regulation of neurogenesis	RAPGEF2
156	0.152988	purine nucleoside triphosphate metabolic process	PARG
157	0.152988	ribonucleoside triphosphate metabolic process	PARG
158	0.152988	regulation of cell projection organization	RAPGEF2
159	0.152988	positive regulation of kinase activity	RAPGEF2
160	0.154515	positive regulation of cell migration	RAPGEF2
161	0.154515	negative regulation of cell cycle	INTS7
162	0.156039	positive regulation of MAPK cascade	RAPGEF2
163	0.159081	response to radiation	INTS7
164	0.159507	response to stress	PARG;FANCC;INTS7

165	0.160598	purine nucleoside monophosphate metabolic process	PARG
166	0.160598	purine ribonucleoside monophosphate metabolic process	PARG
167	0.162113	positive regulation of cell motility	RAPGEF2
168	0.163625	positive regulation of cellular component movement	RAPGEF2
169	0.165135	nucleoside triphosphate metabolic process	PARG
170	0.165135	cellular response to organonitrogen compound	RAPGEF2
171	0.166643	ribonucleoside monophosphate metabolic process	PARG
172	0.169651	negative regulation of cell proliferation	RAPGEF2
173	0.17265	nucleoside monophosphate metabolic process	PARG
174	0.174146	negative regulation of cell differentiation	RAPGEF2
175	0.174146	cellular response to growth factor stimulus	RAPGEF2
176	0.175639	positive regulation of transferase activity	RAPGEF2
177	0.175639	regulation of nervous system development	RAPGEF2
178	0.17713	positive regulation of locomotion	RAPGEF2
179	0.17713	cellular response to organic cyclic compound	RAPGEF2
180	0.178619	epithelial cell differentiation	RAPGEF2
181	0.178619	response to growth factor	RAPGEF2
182	0.18455	central nervous system development	RAPGEF2
183	0.186027	positive regulation of apoptotic process	RAPGEF2
184	0.186027	positive regulation of programmed cell death	RAPGEF2
185	0.187501	neuron projection development	RAPGEF2
186	0.190444	organic hydroxy compound metabolic process	RAPGEF2
187	0.1963	positive regulation of cell death	RAPGEF2
188	0.1963	negative regulation of cellular component organization	RAPGEF2
189	0.1963	cellular response to nitrogen compound	RAPGEF2
190	0.200668	regulation of cell development	RAPGEF2
191	0.201969	intracellular signal transduction	RAPGEF2;ITPR2
192	0.205015	blood vessel morphogenesis	RAPGEF2
193	0.207993	phosphorylation	RAPGEF2;STK10
194	0.209342	DNA repair	FANCC
195	0.212214	purine ribonucleotide metabolic process	PARG
196	0.212214	regulation of MAPK cascade	RAPGEF2
197	0.212783	biological regulation	RAPGEF2;ITPR2;PDE1A;GLRX;STK10;INTS7
198	0.213647	positive regulation of hydrolase activity	RAPGEF2

199	0.216074	regulation of localization	RAPGEF2;STK10
200	0.217933	ribonucleotide metabolic process	PARG
201	0.219357	purine nucleotide metabolic process	PARG
202	0.219357	response to organic cyclic compound	RAPGEF2
203	0.219357	ribose phosphate metabolic process	PARG
204	0.226442	regulation of protein kinase activity	RAPGEF2
205	0.22926	neuron development	RAPGEF2
206	0.23207	carbohydrate metabolic process	PARG
207	0.23207	enzyme linked receptor protein signaling pathway	RAPGEF2
208	0.233471	blood vessel development	RAPGEF2
209	0.236267	generation of precursor metabolites and energy	GLRX
210	0.237662	cell morphogenesis	RAPGEF2
211	0.239054	MAPK cascade	RAPGEF2
212	0.239054	signal transduction by protein phosphorylation	RAPGEF2
213	0.239054	negative regulation of developmental process	RAPGEF2
214	0.241832	purine-containing compound metabolic process	PARG
215	0.243218	vasculature development	RAPGEF2
216	0.243941	cellular process	RAPGEF2;PARG;FANCC;ITPR2;PDE1A;GLRX;STK10;INTS7
217	0.245983	cardiovascular system development	RAPGEF2
218	0.251486	response to organonitrogen compound	RAPGEF2
219	0.252856	positive regulation of cell differentiation	RAPGEF2
220	0.25559	tube morphogenesis	RAPGEF2
221	0.25559	regulation of kinase activity	RAPGEF2
222	0.258315	G protein-coupled receptor signaling pathway	RAPGEF2
223	0.261032	nucleotide metabolic process	PARG
224	0.26374	regulation of cellular component biogenesis	RAPGEF2
225	0.26509	nucleoside phosphate metabolic process	PARG
226	0.26509	neuron differentiation	RAPGEF2
227	0.26509	cellular component morphogenesis	RAPGEF2
228	0.269129	cellular homeostasis	GLRX
229	0.270471	positive regulation of protein phosphorylation	RAPGEF2
230	0.279805	regulation of anatomical structure morphogenesis	RAPGEF2
231	0.281129	positive regulation of phosphorylation	RAPGEF2
232	0.283457	signal transduction	RAPGEF2;ITPR2;PDE1A

233	0.286407	regulation of transferase activity	RAPGEF2
234	0.286407	epithelium development	RAPGEF2
235	0.286407	response to nitrogen compound	RAPGEF2
236	0.292957	positive regulation of intracellular signal transduction	RAPGEF2
237	0.302038	positive regulation of phosphorus metabolic process	RAPGEF2
238	0.302038	positive regulation of phosphate metabolic process	RAPGEF2
239	0.302038	nucleobase-containing small molecule metabolic process	PARG
240	0.303327	tube development	RAPGEF2
241	0.303327	regulation of cell cycle	INTS7
242	0.309741	generation of neurons	RAPGEF2
243	0.309741	cellular response to oxygen-containing compound	RAPGEF2
244	0.313564	positive regulation of cellular component organization	RAPGEF2
245	0.314385	signaling	RAPGEF2;ITPR2;PDE1A
246	0.317369	circulatory system development	RAPGEF2
247	0.318633	positive regulation of protein modification process	RAPGEF2
248	0.319895	response to abiotic stimulus	INTS7
249	0.322413	negative regulation of multicellular organismal process	RAPGEF2
250	0.324923	plasma membrane bounded cell projection organization	RAPGEF2
251	0.325382	cell communication	RAPGEF2;ITPR2;PDE1A
252	0.328672	negative regulation of cellular biosynthetic process	RAPGEF2
253	0.329918	drug metabolic process	PARG
254	0.333643	DNA metabolic process	FANCC
255	0.338582	negative regulation of biosynthetic process	RAPGEF2
256	0.338582	cell projection organization	RAPGEF2
257	0.339812	neurogenesis	RAPGEF2
258	0.351999	positive regulation of developmental process	RAPGEF2
259	0.360413	cellular response to endogenous stimulus	RAPGEF2
260	0.360948	cellular aromatic compound metabolic process	RAPGEF2;PARG;FANCC
261	0.361607	positive regulation of catalytic activity	RAPGEF2
262	0.361607	regulation of hydrolase activity	RAPGEF2
263	0.386236	regulation of protein phosphorylation	RAPGEF2
264	0.388538	organophosphate metabolic process	PARG

265	0.389686	regulation of immune system process	STK10
266	0.390832	regulation of cell proliferation	RAPGEF2
267	0.390832	response to oxygen-containing compound	RAPGEF2
268	0.395398	response to endogenous stimulus	RAPGEF2
269	0.396315	organic cyclic compound metabolic process	RAPGEF2;PARG;FANCC
270	0.396535	positive regulation of signal transduction	RAPGEF2
271	0.396535	regulation of cell differentiation	RAPGEF2
272	0.399934	regulation of apoptotic process	RAPGEF2
273	0.405561	carbohydrate derivative metabolic process	PARG
274	0.406681	regulation of programmed cell death	RAPGEF2
275	0.41447	positive regulation of cellular protein metabolic process	RAPGEF2
276	0.419978	regulation of phosphorylation	RAPGEF2
277	0.424352	positive regulation of cell communication	RAPGEF2
278	0.424352	regulation of cell death	RAPGEF2
279	0.424352	positive regulation of multicellular organismal process	RAPGEF2
280	0.425441	positive regulation of signaling	RAPGEF2
281	0.436232	positive regulation of protein metabolic process	RAPGEF2
282	0.442621	positive regulation of molecular function	RAPGEF2
283	0.445761	negative regulation of cellular process	RAPGEF2;INTS7
284	0.445791	regulation of multicellular organismal development	RAPGEF2
285	0.448946	regulation of intracellular signal transduction	RAPGEF2
286	0.449994	nervous system development	RAPGEF2
287	0.457282	tissue development	RAPGEF2
288	0.458317	cell development	RAPGEF2
289	0.464486	regulation of phosphate metabolic process	RAPGEF2
290	0.464486	regulation of phosphorus metabolic process	RAPGEF2
291	0.469143	cellular protein modification process	RAPGEF2;STK10
292	0.469143	protein modification process	RAPGEF2;STK10
293	0.472616	cell proliferation	RAPGEF2
294	0.473625	regulation of protein modification process	RAPGEF2
295	0.480638	homeostatic process	GLRX
296	0.49446	cellular metabolic process	RAPGEF2;PARG;FANCC;GLRX;STK10
297	0.503795	macromolecule modification	RAPGEF2;STK10
298	0.522879	oxidation-reduction process	GLRX
299	0.524858	negative regulation of biological process	RAPGEF2;INTS7
300	0.527483	apoptotic process	RAPGEF2
301	0.536575	programmed cell death	RAPGEF2

302	0.537476	positive regulation of response to stimulus	RAPGEF2
303	0.542848	cell surface receptor signaling pathway	RAPGEF2
304	0.548791	macromolecule metabolic process	RAPGEF2;PARG;FANCC;STK10
305	0.549047	cellular response to organic substance	RAPGEF2
306	0.553429	negative regulation of cellular metabolic process	RAPGEF2
307	0.554301	cell death	RAPGEF2
308	0.556908	regulation of cellular component organization	RAPGEF2
309	0.558639	regulation of developmental process	RAPGEF2
310	0.568899	anatomical structure morphogenesis	RAPGEF2
311	0.571431	regulation of catalytic activity	RAPGEF2
312	0.606322	immune system process	STK10
313	0.60866	negative regulation of metabolic process	RAPGEF2
314	0.617323	organonitrogen compound metabolic process	RAPGEF2;PARG;STK10
315	0.634328	regulation of cellular protein metabolic process	RAPGEF2
316	0.638739	nucleobase-containing compound metabolic process	PARG;FANCC
317	0.640149	response to organic substance	RAPGEF2
318	0.647311	regulation of multicellular organismal process	RAPGEF2
319	0.64802	animal organ development	RAPGEF2
320	0.653649	small molecule metabolic process	PARG
321	0.656224	nitrogen compound metabolic process	RAPGEF2;PARG;FANCC;STK10
322	0.65851	cellular response to chemical stimulus	RAPGEF2
323	0.659888	positive regulation of nitrogen compound metabolic process	RAPGEF2
324	0.660148	cellular macromolecule metabolic process	RAPGEF2;FANCC;STK10
325	0.660575	regulation of protein metabolic process	RAPGEF2
326	0.662773	heterocycle metabolic process	PARG;FANCC
327	0.666368	metabolic process	RAPGEF2;PARG;FANCC;GLRX;STK10
328	0.6762	cellular protein metabolic process	RAPGEF2;STK10
329	0.676705	cellular component assembly	RAPGEF2
330	0.678019	positive regulation of cellular metabolic process	RAPGEF2
331	0.679327	positive regulation of macromolecule metabolic process	RAPGEF2
332	0.708772	cellular component biogenesis	RAPGEF2
333	0.712355	positive regulation of metabolic process	RAPGEF2
334	0.71354	regulation of signal transduction	RAPGEF2
335	0.726872	regulation of cellular biosynthetic process	RAPGEF2
336	0.735968	primary metabolic process	RAPGEF2;PARG;FANCC;STK10

337	0.736873	localization	RAPGEF2;STK10
338	0.737472	regulation of biosynthetic process	RAPGEF2
339	0.741285	regulation of cell communication	RAPGEF2
340	0.741826	cell differentiation	RAPGEF2
341	0.743443	regulation of signaling	RAPGEF2
342	0.746117	regulation of molecular function	RAPGEF2
343	0.751301	protein metabolic process	RAPGEF2;STK10
344	0.755029	aromatic compound biosynthetic process	RAPGEF2
345	0.764166	cellular developmental process	RAPGEF2
346	0.774531	cellular nitrogen compound metabolic process	PARG;FANCC
347	0.775883	organic cyclic compound biosynthetic process	RAPGEF2
348	0.776836	regulation of biological quality	GLRX
349	0.782481	response to chemical	RAPGEF2
350	0.790263	system development	RAPGEF2
351	0.791028	organic substance metabolic process	RAPGEF2;PARG;FANCC;STK10
352	0.809284	regulation of response to stimulus	RAPGEF2
353	0.843551	multicellular organism development	RAPGEF2
354	0.855018	nucleic acid metabolic process	FANCC
355	0.858885	positive regulation of cellular process	RAPGEF2
356	0.876901	regulation of nitrogen compound metabolic process	RAPGEF2
357	0.877183	anatomical structure development	RAPGEF2
358	0.886452	regulation of primary metabolic process	RAPGEF2
359	0.892117	regulation of cellular metabolic process	RAPGEF2
360	0.892369	regulation of macromolecule metabolic process	RAPGEF2
361	0.899207	developmental process	RAPGEF2
362	0.900622	positive regulation of biological process	RAPGEF2
363	0.915483	regulation of metabolic process	RAPGEF2
364	0.921776	cellular component organization	RAPGEF2
365	0.925861	cellular biosynthetic process	RAPGEF2
366	0.929585	cellular component organization or biogenesis	RAPGEF2
367	0.931639	organic substance biosynthetic process	RAPGEF2
368	0.935909	biosynthetic process	RAPGEF2
369	0.940843	multicellular organismal process	RAPGEF2
370	0.001538	beta-1 adrenergic receptor binding	RAPGEF2
371	0.003074	calmodulin-dependent cyclic-nucleotide phosphodiesterase activity	PDE1A
372	0.003074	poly(ADP-ribose) glycohydrolase activity	PARG
373	0.003074	Rap guanyl-nucleotide exchange factor activity	RAPGEF2

374	0.003074	calcium- and calmodulin-regulated 3',5'-cyclic-GMP phosphodiesterase activity	PDE1A
375	0.007671	cAMP binding	RAPGEF2
376	0.007671	adrenergic receptor binding	RAPGEF2
377	0.009199	WW domain binding	RAPGEF2
378	0.010725	3',5'-cyclic-GMP phosphodiesterase activity	PDE1A
379	0.012249	cyclic-nucleotide phosphodiesterase activity	PDE1A
380	0.012249	3',5'-cyclic-nucleotide phosphodiesterase activity	PDE1A
381	0.013772	protein disulfide oxidoreductase activity	GLRX
382	0.016811	PDZ domain binding	RAPGEF2
383	0.018327	Ras guanyl-nucleotide exchange factor activity	RAPGEF2
384	0.021354	cyclic nucleotide binding	RAPGEF2
385	0.022865	disulfide oxidoreductase activity	GLRX
386	0.033385	oxidoreductase activity, acting on a sulfur group of donors	GLRX
387	0.03488	phosphoric diester hydrolase activity	PDE1A
388	0.045292	hydrolase activity, hydrolyzing O-glycosyl compounds	PARG
389	0.049726	electron transfer activity	GLRX
390	0.0512	guanyl-nucleotide exchange factor activity	RAPGEF2
391	0.055611	GTPase activator activity	RAPGEF2
392	0.055611	hydrolase activity, acting on glycosyl bonds	PARG
393	0.060004	GTPase regulator activity	RAPGEF2
394	0.067289	Ras GTPase binding	RAPGEF2
395	0.067289	small GTPase binding	RAPGEF2
396	0.071638	G protein-coupled receptor binding	RAPGEF2
397	0.073083	calmodulin binding	PDE1A
398	0.075969	nucleoside-triphosphatase regulator activity	RAPGEF2
399	0.093127	protein serine/threonine kinase activity	STK10
400	0.104417	GTPase binding	RAPGEF2
401	0.110018	phosphoric ester hydrolase activity	PDE1A
402	0.118148	adenyl ribonucleotide binding	RAPGEF2;STK10
403	0.118659	adenyl nucleotide binding	RAPGEF2;STK10
404	0.122513	enzyme activator activity	RAPGEF2
405	0.123893	protein kinase activity	STK10
406	0.129783	protein binding	RAPGEF2;MFAP2;PDE1A;STK10
407	0.136688	catalytic activity	PARG;PDE1A;GLRX;STK10

408	0.151102	phosphotransferase activity, alcohol group as acceptor	STK10
409	0.17236	protein domain specific binding	RAPGEF2
410	0.173673	kinase activity	STK10
411	0.184917	purine ribonucleotide binding	RAPGEF2;STK10
412	0.185496	purine nucleotide binding	RAPGEF2;STK10
413	0.186075	ribonucleotide binding	RAPGEF2;STK10
414	0.222323	transferase activity, transferring phosphorus-containing groups	STK10
415	0.227574	hydrolase activity	PARG;PDE1A
416	0.23242	carbohydrate derivative binding	RAPGEF2;STK10
417	0.240858	hydrolase activity, acting on ester bonds	PDE1A
418	0.240942	nucleotide binding	RAPGEF2;STK10
419	0.240942	nucleoside phosphate binding	RAPGEF2;STK10
420	0.254215	enzyme regulator activity	RAPGEF2
421	0.270931	protein homodimerization activity	STK10
422	0.311136	small molecule binding	RAPGEF2;STK10
423	0.32183	anion binding	RAPGEF2;STK10
424	0.365863	oxidoreductase activity	GLRX
425	0.37632	protein dimerization activity	STK10
426	0.382525	signaling receptor binding	RAPGEF2
427	0.430337	enzyme binding	RAPGEF2
428	0.451197	identical protein binding	STK10
429	0.460465	molecular function regulator	RAPGEF2
430	0.462303	ATP binding	STK10
431	0.504309	ion binding	RAPGEF2;PDE1A;STK10
432	0.511691	catalytic activity, acting on a protein	STK10
433	0.523428	drug binding	STK10
434	0.530029	transferase activity	STK10
435	0.559576	purine ribonucleoside triphosphate binding	STK10
436	0.635012	heterocyclic compound binding	RAPGEF2;STK10
437	0.643237	organic cyclic compound binding	RAPGEF2;STK10
438	0.862881	metal ion binding	PDE1A
439	0.865215	cation binding	PDE1A
440	0.869922	binding	RAPGEF2;MFAP2;PDE1A;STK10
441	0.004422	Fanconi anaemia nuclear complex	FANCC
442	0.008827	microfibril	MFAP2
443	0.016132	extracellular matrix component	MFAP2
444	0.036341	rough endoplasmic reticulum	ITPR2
445	0.040509	nucleoplasm part	FANCC;INTS7
446	0.040625	secretory granule membrane	ITPR2
447	0.070157	transport vesicle membrane	ITPR2
448	0.072928	neuronal cell body	RAPGEF2
449	0.088046	extracellular matrix	MFAP2

450	0.098907	cell body	RAPGEF2
451	0.100257	secretory granule	ITPR2
452	0.10698	late endosome	RAPGEF2
453	0.117647	cell-cell junction	RAPGEF2
454	0.117647	transport vesicle	ITPR2
455	0.137354	somatodendritic compartment	RAPGEF2
456	0.143839	secretory vesicle	ITPR2
457	0.155743	cytoplasmic vesicle	RAPGEF2;ITPR2
458	0.155743	intracellular vesicle	RAPGEF2;ITPR2
459	0.158924	nucleoplasm	FANCC;INTS7
460	0.169643	vesicle	RAPGEF2;ITPR2
461	0.19054	cytoplasmic vesicle membrane	ITPR2
462	0.193001	vesicle membrane	ITPR2
463	0.210048	nuclear body	INTS7
464	0.231512	perinuclear region of cytoplasm	RAPGEF2
465	0.263906	supramolecular fiber	MFAP2
466	0.266175	supramolecular complex	MFAP2
467	0.266175	supramolecular polymer	MFAP2
468	0.267826	nuclear lumen	FANCC;INTS7
469	0.281886	endosome	RAPGEF2
470	0.282998	synapse	RAPGEF2
471	0.286322	neuron projection	RAPGEF2
472	0.300576	chromosome	INTS7
473	0.303831	cytoplasmic vesicle part	ITPR2
474	0.334629	cell junction	RAPGEF2
475	0.345928	nuclear part	FANCC;INTS7
476	0.371229	neuron part	RAPGEF2
477	0.373162	plasma membrane	RAPGEF2;STK10
478	0.379124	extracellular region part	MFAP2
479	0.387902	cell periphery	RAPGEF2;STK10
480	0.411931	membrane-enclosed lumen	FANCC;INTS7
481	0.411931	organelle lumen	FANCC;INTS7
482	0.411931	intracellular organelle lumen	FANCC;INTS7
483	0.436578	whole membrane	ITPR2
484	0.459816	endomembrane system	RAPGEF2;ITPR2
485	0.475984	plasma membrane bounded cell projection	RAPGEF2
486	0.493243	cytoplasm	RAPGEF2;FANCC;ITPR2;GLRX;INTS7
487	0.496016	cell projection	RAPGEF2
488	0.498474	endoplasmic reticulum	ITPR2
489	0.529496	bounding membrane of organelle	ITPR2
490	0.588711	extracellular region	MFAP2
491	0.607314	protein-containing complex	RAPGEF2;FANCC
492	0.690967	nucleus	FANCC;INTS7
493	0.700581	membrane	RAPGEF2;ITPR2;STK10

494	0.712463	intracellular organelle part	FANCC;ITPR2;INTS7
495	0.721756	membrane-bounded organelle	RAPGEF2;FANCC;ITPR2;INTS7
496	0.74112	organelle part	FANCC;ITPR2;INTS7
497	0.756405	organelle membrane	ITPR2
498	0.758996	cell part	RAPGEF2;FANCC;ITPR2;GLRX;STK10;INTS7
499	0.769384	cell	RAPGEF2;FANCC;ITPR2;GLRX;STK10;INTS7
500	0.824487	intracellular	RAPGEF2;FANCC;ITPR2;GLRX;INTS7
501	0.824487	intracellular part	RAPGEF2;FANCC;ITPR2;GLRX;INTS7
502	0.833828	non-membrane-bounded organelle	INTS7
503	0.833828	intracellular non-membrane-bounded organelle	INTS7
504	0.83676	intracellular organelle	RAPGEF2;FANCC;ITPR2;INTS7
505	0.855444	intracellular membrane-bounded organelle	FANCC;ITPR2;INTS7
506	0.857109	organelle	RAPGEF2;FANCC;ITPR2;INTS7
507	0.86599	integral component of membrane	ITPR2
508	0.875448	intrinsic component of membrane	ITPR2
509	0.931253	cytoplasmic part	RAPGEF2;ITPR2
510	0.945497	membrane part	ITPR2
