# Supplementary material for: Whole-genome resequencing reveals domestication and signatures of selection in Ujimqin, Sunit, and Wu Ranke Mongolian sheep breeds
Source: Anim Biosci. 2022 Apr 29;35(9):1303–13. doi: 10.5713/ab.21.0569 (PMC9449395; doi:10.5713/ab.21.0569)
Supplement: Supplementary Table S4. — KEGG between UJMQ and WRK [file ab-21-0569-suppl6.pdf]

Whole-genome resequencing reveals domestication and signatures of selection in Ujimqin, Sunit, and Wu Ranke Mongolian sheep breeds

Hanning *Wang*[1a], Liang *Zhong*[2a], Yanbing *Dong*[1], Lingbo *Meng*[1], Cheng *Ji*[1], Hui *Luo*[1], Mengrong *Fu*[1], Zhi *Qi*[1*], and Lan *Mi*[1*]

***Corresponding Author: Lan Mi**

E-mail: lanmi_90@126.com

Zhi Qi

E-mail: qizhi@imu.edu.cn

[1] State Key Laboratory of Reproductive Regulation and Breeding of Grassland Livestock, School of Life Sciences, Inner Mongolia University, Hohhot 020020, China

[2] Hebei Provincial Key Laboratory of Basic Medicine for Diabetes, The Shijiazhuang Second Hospital, Shijiazhuang 050051, China

[a] These authors contributed equally to this work.

Table S4 KEGG between UJMQ and WRK

	P-value	Term	Symbols
1	0.004522	Long-term potentiation	ITPR2;GRIN2B
2	0.005766	Phosphatidylinositol signaling system	ITPR2;DGKB
3	0.028939	Calcium signaling pathway	ITPR2;PDE1A
4	0.029241	Alzheimer's disease	ITPR2;GRIN2B
5	0.060525	Neuroactive ligand-receptor interaction	GRIN2B;GRID1
6	0.074186	Glycerolipid metabolism	DGKB
7	0.079904	Amyotrophic lateral sclerosis (ALS)	GRIN2B
8	0.094061	p53 signaling pathway	CHEK2
9	0.102462	Melanoma	MITF
10	0.103856	Gastric acid secretion	ITPR2
11	0.105247	Long-term depression	ITPR2
12	0.112176	Salivary secretion	ITPR2
13	0.117685	Glycerophospholipid metabolism	DGKB
14	0.129969	Gap junction	ITPR2
15	0.136727	Melanogenesis	MITF
16	0.139417	Pancreatic secretion	ITPR2
17	0.139417	GnRH signaling pathway	ITPR2
18	0.163296	Vascular smooth muscle contraction	ITPR2
19	0.169824	Oocyte meiosis	ITPR2
20	0.172422	Axon guidance	PLXNA2
21	0.175013	Osteoclast differentiation	MITF
22	0.18146	Cell cycle	CHEK2
23	0.220437	Purine metabolism	PDE1A
24	0.253031	Systemic lupus erythematosus	GRIN2B
25	0.262453	Huntington's disease	GRIN2B
26	0.339436	MAPK signaling pathway	RAPGEF2
27	0.388689	Pathways in cancer	MITF
28	0.845593	Metabolic pathways	DGKB
